# Identification of key biomarkers for myocardial infarction by multi-omics analysis and machine learning

**DOI:** 10.3389/fimmu.2026.1711521

**Published:** 2026-04-13

**Authors:** Jiacheng Wu, Yulu Yang, Jianwu Huang, Xuehan Li, Qian Ma, Hao Chen, Yalei Wang, Erha Lama, Zhihua Qiu, Zihua Zhou

**Affiliations:** 1 Department of Cardiology, Union Hospital, Tongji Medical College, Huazhong University of Science and Technology, Wuhan, China; 2 Hubei Key Laboratory of Biological Targeted Therapy, Union Hospital, Tongji Medical College, Huazhong University of Science and Technology, Wuhan, China; 3 Hubei Provincial Engineering Research Center of Immunological Diagnosis and Therapy for Cardiovascular Diseases, Union Hospital, Tongji Medical College, Huazhong University of Science and Technology, Wuhan, China; 4 Department of Cardiology, Renmin Hospital of Wuhan University, Wuhan, Hubei, China

**Keywords:** bioinformatics, biomarkers, immune infiltration, machine learning, myocardial infarction, TWAS

## Abstract

**Background:**

Acute myocardial infarction (AMI) is one of the leading causes of mortality worldwide. Despite extensive research, only a limited number of genes have been identified as reliable biomarkers for the diagnosis and treatment of AMI. This study aims to identify novel biomarkers and therapeutic targets for AMI by integrating multi-omics data and machine learning.

**Methods:**

We obtained the GWAS dataset I9_MI_STRICT from the FinnGen database and the eQTL dataset of peripheral blood from the GTEx database. Using these datasets, we identified genes significantly associated with AMI through transcriptome-wide association studies (TWAS). Functional enrichment analysis was performed using Gene Ontology (GO) and Kyoto Encyclopedia of Genes and Genomes (KEGG) pathways. Additionally, we downloaded three AMI peripheral blood gene expression microarray datasets (GSE66360, GSE48060, GSE60993) from the Gene Expression Omnibus (GEO) database. Key genes were further identified by combining the risk prediction model constructed by 12 machine learning methods(dataset GSE66360 as the training set, dataset GSE48060 and dataset GSE60993 as the validation set) and Bayesian colocalization analysis. To explore the potential mechanisms of these key genes in AMI, we conducted immunoinfiltration analysis, single-gene Gene Set Enrichment Analysis (GSEA), and Gene Set Variation Analysis (GSVA). Finally, the expression of key genes was validated using real-time quantitative PCR (RT-qPCR) and western blot.

**Results:**

We identified several key genes: *LIPA, PECAM1, SMARCA4, HP, RTN2, CFDP1, XPO6*, and *FES*. Receiver operating characteristic (ROC) analysis demonstrated that these genes exhibited excellent diagnostic performance. Enrichment analysis revealed their primary involvement in lipid metabolism, immune system processes, gene transcription regulation, and ion channel regulation. Furthermore, immunoinfiltration analysis showed that *PECAM1, HP, RTN2, CFDP1*, and *FES* were significantly correlated with various immune cell types. qRT-PCR and western blot analysis revealed that the mRNA expression of *LIPA, RTN2*, and *PECAM1* was upregulated in the AMI group, while *CFDP1* and *XPO6* showed downregulation compared to the control group.

**Conclusions:**

This study identified nine key genes as potential novel targets for the diagnosis and treatment of AMI.

## 1 Introduction

Acute myocardial infarction (AMI) is characterized by irreversible myocardial necrosis resulting from the acute occlusion of coronary arteries. It is one of the leading causes of cardiovascular disease-related morbidity and mortality worldwide (1). Although early diagnosis and treatment (particularly percutaneous coronary intervention), have been shown to reduce cardiomyocyte death, decrease mortality, and improve prognosis, hospital admission and mortality rates remain high (2). Furthermore, heart failure and sudden cardiac death following AMI continue to pose significant challenges (3). The underlying regulatory mechanisms of AMI are not yet fully understood, making it essential to explore the pathogenic pathways and identify novel therapeutic targets for AMI.

Immune and inflammation play a key role in the onset and progression of AMI (4). The initial acute inflammatory response in AMI is triggered by damaged cardiomyocytes. Following myocardial injury, immune cells, such as neutrophils and macrophages, are recruited to the ischemic heart tissue, where they coordinate the inflammatory response (5). Based on these processes, various machine learning algorithms have been integrated to identify novel diagnostic biomarkers for AMI and explore potential associations between these biomarkers and immune cell infiltration (6, 7). In addition, there is increasing evidence that genetic factors play a crucial role in the development of AMI. Genome-wide association studies (GWAS) have identified several susceptibility loci for AMI (8). However, research integrating GWAS, eQTL, GEO data, and machine learning algorithms to investigate AMI-related biomarkers and pathogenesis remains limited.

**Figure 1:**
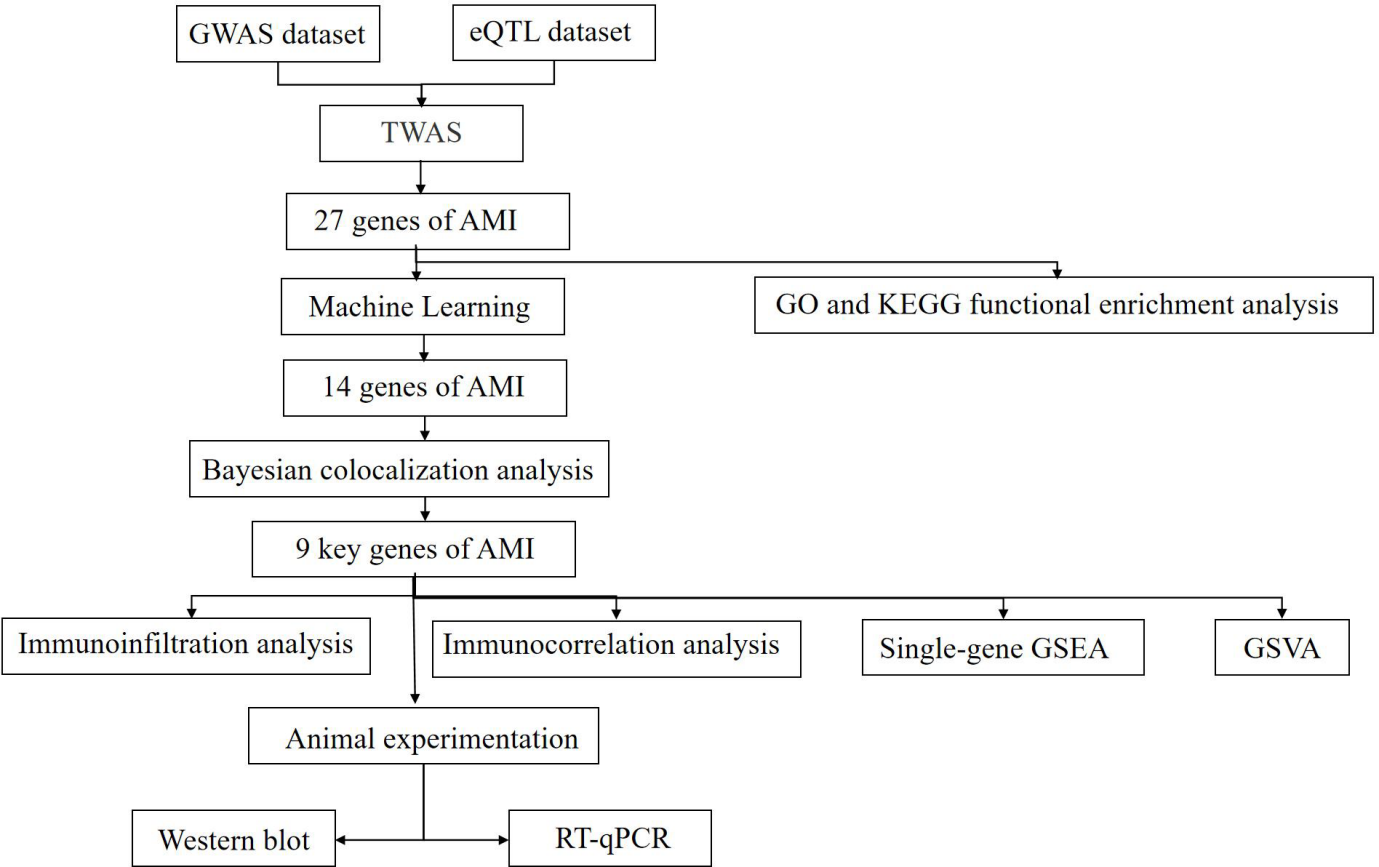
Workflow of the study.

In this study, we utilized the GWAS and eQTL datasets to identify genes significantly associated with AMI by transcriptome-wide association studies (TWAS). Additionally, we constructed a risk prediction model and Bayesian colocalization analysis to further identify key genes involved in AMI. To explore the molecular mechanisms underlying the role of these key genes in AMI, we applied bioinformatics techniques such as enrichment analysis, immunoinfiltration analysis, single-gene Gene Set Enrichment Analysis (GSEA), and Gene Set Variation Analysis (GSVA). Our study provides potential biomarkers, pathogenesis, and therapeutic strategies for AMI. The overall workflow of this study is depicted in **Figure 1**.

## 2 Materials and methods

### 2.1 GWAS data for AMI

In this study, we utilized the I9_MI_STRICT dataset from the 11th edition of the FinnGen Project, a large-scale research initiative aimed at uncovering genetic risk factors for various diseases. This dataset specifically focuses on strictly defined cases of myocardial infarction and includes 28,546 cases and 378,019 controls. By integrating genotype data with electronic health records from the Finnish population, the I9_MI_STRICT dataset offers a valuable resource for studying the genetic underpinnings of myocardial infarction. The data can be accessed via Google Cloud Storage (https://storage.googleapis.com/finngen-public-data-r11/summary_stats/finngen_R11_I9_MI_STRICT.gz). With its high-quality data, the FinnGen project has significant potential for GWAS to identify genetic variants associated with myocardial infarction, thus contributing to both basic research and clinical advancements in cardiovascular disease.

### 2.2 eQTL data

The GTEx database (https://gtexportal.org/home/) was used to study the effects of tissue-specific gene expression and splicing genetic mutations (9). The latest, 8th edition of the database contains genomic and transcriptomic data from 17,382 samples across 54 tissues and 2 cell lines obtained from 838 posthumous donors. In alignment with the information used in TWAS and Summary-data-based Mendelian Randomization analysis, this study primarily utilized the eQTL data from peripheral blood in the GTEx v8 version. The results of QTL analyses and related visualizations in this database are freely accessible (10).

### 2.3 Screening and processing of gene expression datasets

We downloaded gene expression profile microarrays (GSE66360, GSE48060, GSE60993) from the Gene Expression Omnibus (GEO) database, which include peripheral blood samples from both control and myocardial infarction patients. The details of these datasets are provided in **Table 1**. To standardize the data, we converted probe names to gene symbols using the corresponding platform annotation files. The R package limma was employed to perform background correction and normalization, thereby eliminating batch effects. For the subsequent analysis, GSE66360 was used as the training set, while GSE48060 and GSE60993 were used as validation sets. Detailed demographic and clinical characteristics of the samples are available in the corresponding GEO dataset metadata and original submissions.

**Table 1:** Summary of AMI transcriptome sequencing data.

Dataset	Platform	Tissue	AMI	Control	Category
GSE48060	GPL570	blood	31	21	validation
GSE60993	GPL6884	blood	17	7	validation
GSE66360	GPL570	blood	49	50	training

### 2.4 Transcriptome-wide association study

We employed the FUSION tool (http://gusevlab.org/projects/fusion/) to conduct TWAS for AMI (11). Based on eQTLs’ predictive model and GWAS statistics, this tool tests the association between gene expression and disease characteristics. Our TWAS analysis focused on blood tissue, leveraging aggregated GWAS data on AMI, FUSION’s blood tissue gene expression prediction model, and data from the 1000 Genomes Project as a reference panel for linkage disequilibrium. A variety of predictive models, including top1, BLUP, Lasso, ENet, and BSLMM, were used to identify the most predictive model for estimating gene influence. By combining the genetic effects and gene weights for AMI, we performed TWAS specifically for AMI. This analysis was limited to autosomal chromosomes and conducted according to FUSION’s default settings. Only genes regulated by cis-eQTLs (significant cis-SNP heritability (P < 0.01))were included in the gene expression association analysis. The gene expression prediction model was constructed using several methods, including the best-performing cis-eQTL model and elastic net regression. The most accurate model was selected for expression prediction. Using this established gene expression prediction model, we performed TWAS for all significant genetic traits and calculated the TWAS Z-scores for each trait. Significant TWAS results were corrected for multiple testing using the Bonferroni method (12). Genes lacking significant GWAS association signals within a 500 kb window were considered newly identified susceptibility genes.

### 2.5 Bayesian colocalization analysis

We used Bayesian colocalization analysis to assess the probability that two traits share the same causal variation (13), employing the “coloc” package with default parameters (https://github.com/chr1swallace/coloc). As described earlier, Bayesian colocalization calculates the posterior probabilities for five hypotheses regarding whether a single variation is shared between two traits. These five hypotheses are as follows: H0: Neither phenotype (exposure nor outcome) is associated with SNPs. H1: The first phenotype (exposure) is associated with SNPs, but the second phenotype (outcome) is not. H2: The second phenotype (outcome) is associated with SNPs, but the first phenotype (exposure) is not. H3: Both phenotypes are associated with SNPs, but the two relationships are independent. H4: Both phenotypes are associated with SNPs, and both relationships share the same causal SNP (14). In this study, we focused on hypothesis H4 (PPH 4), where both genes and AMI are associated with SNPs, and the shared causal SNP drives both relationships. We considered a gene to have Strong colocalization evidence if gene-based PPH 4>0.8 (15).

### 2.6 Functional enrichment analysis

GO and KEGG enrichment analyses were performed on the pathogenic genes associated with AMI (16, 17). The background gene set for GO and KEGG enrichment analyses was defined as all annotated protein-coding genes in the human genome to ensure an unbiased functional characterization. The filtration criteria for enrichment analysis were as follows: p< 0.05 was considered significant. The org.Hs.eg.db package was used for gene ID conversion, while the clusterProfiler package was employed for GO functional enrichment analysis, which included the biological process (BP), cellular component (CC), and molecular function (MF) categories. Additionally, KEGG pathway enrichment analysis was conducted to explore the biological pathways involved in the pathogenic genes of AMI.

### 2.7 single-gene GSEA

Single-gene GSEA is a bioinformatics method that utilizes gene expression profile data to explore the regulatory pathways and molecular functions associated with a specific gene. For each target gene, all other genes were ranked according to their Spearman correlation coefficients with the expression level of the target gene across samples. This correlation-based ranked gene list was then used as input for GSEA to identify biological pathways positively or negatively associated with the target gene. Unlike traditional GSEA, which analyzes gene sets, single-gene GSEA focuses on individual genes. It examines the correlation between the expression of a target gene and other genes, and investigates the enrichment of these associated genes in relevant regulatory pathways and molecular functions.

### 2.8 Development and validation of predictive models based on diagnostic markers

In this study, we adopted a classification prediction model developed by Aierpati Maimaiti et al., which incorporates 12 commonly used machine learning algorithms and 101 parameter combinations (18). The algorithms include Lasso (Lasso regression), Ridge (Ridge regression), Enet (Elastic network), Stepglm (Stepwise generalized linear model), SVM (Support Vector Machine), glmBoost (Generalized Linear Model Boosting), LDA (Linear Discriminant Analysis), plsRglm (Partial Least Squares Regression and Generalized Linear Model), RandomForest (Random Forest), GBM (Gradient Boosting Machine), XGBoost (Extreme Gradient Boosting), and NaiveBayes (Naive Bayes). Model performance was evaluated primarily by calculating the area under the ROC curve (AUC) for each model and its respective gene. The results were then visually displayed using heat maps.

### 2.9 Analysis of immune cell infiltration

We performed immunoinfiltration analysis using the R package IOBR, which allows batch analysis of immune characteristics and their association with clinical phenotypes. IOBR supports 8 common immune infiltration algorithms, and we specifically utilized the CIBERSORT algorithm for this analysis (http://cibersort.stanford.edu/). CIBERSORT is a method designed to characterize the cellular composition of complex tissues using gene expression profiles (19). The CIBERSORT software package can analyze the expression of 22 different immune cell types in patient tissues.

### 2.10 Gene set variation analysis

GSVA was used to identify the potential functions of key gene. We retrieved a subset of external reference genes from the Molecular Signature Database (MSigDB) and selected the C5: Ontology gene set for analysis. The significance threshold for enrichment was set at p< 0.05. From this, we extracted 15 gene sets, each containing 25 genes associated with macrophages. The R package “GSVA” was then used to analyze the correlation between these key genes and macrophage regulation. Heat maps were generated using the “ggplot2” and “ggcor” R packages.

### 2.11 Single-cell RNA sequencing analysis

Single-cell transcriptome data analysis was performed using the Seurat R package ecosystem. Initial gene expression matrices were imported via the Read10X function, followed by stringent quality control (QC). Only high-quality cells with 200–10,000 detected genes and a total UMI count exceeding 1,000 were retained. To exclude damaged or dying cells, a threshold of <20% mitochondrial and ribosomal gene content was applied. Additionally, only genes expressed in at least 5 cells were included for downstream analysis.

Data were normalized using the LogNormalize method (scale factor = 10,000), and the top 2,500 highly variable genes (HVGs) were identified via the variance-stabilizing transformation (VST) algorithm. Following linear regression to mitigate unwanted sources of variation, dimensionality reduction was performed using Principal Component Analysis (PCA). The top 30 principal components (PCs) were then utilized for two-dimensional visualization via the UMAP algorithm. To determine optimal clustering granularity, we employed the Clustree package for iterative evaluation (resolution range: 0–1.0) and validated clustering stability using silhouette scores. Cluster-specific marker genes were identified using the Wilcoxon rank-sum test (|log2FC| > 1.0, adjusted P < 0.05). Automated cell-type annotation was performed using SingleR with the Human Primary Cell Atlas (HPCA) as a reference, followed by rigorous manual curation based on canonical marker expression.

### 2.12 Acute myocardial infarction model

Male C57BL/6 mice aged 8 weeks were randomly divided into two groups: 1) Control group; 2) Myocardial infarction group. First, the skin and the third and fourth ribs of the mice were incised to fully expose the heart and the left anterior descending artery. The blood vessels were then ligated approximately 2 mm from the lower margin of the left atrial appendage. Successful ligation was confirmed when the distal area of the ligature turned white. Mice in the control group underwent the same procedure, but without vascular ligation.

### 2.13 Total RNA isolation and real-time quantitative PCR analysis

Total RNA extraction from heart was carried out using RNAiso Plus (Takara, Shiga, Japan) following the manufacturer’s protocol. The expression of relevant genes was analyzed through real-time quantitative PCR (RT-qPCR) using a Step One Real-Time PCR machine (Applied Biosystems, California, USA) and TB Green Premix Ex Taq (Takara, Shiga, Japan). The primer information was shown in **Table 2**-2.

**Table 2:** The primer sequences of nine biomarkers used in the real-time quantitative PCR (RT-qPCR).

Primer	Sequences(5’-3’)	Sequences(5’-3’)
*iNOS*	AACCCCTTGTGCTGTTCTCAGCC	GTGGACGGGTCGATGTCACATGC
*IL-6*	TGATGCTGGTGACAACCACGGC	TAAGCCTCCGACTTGTGAAGTGGTA
*IL-1β*	AAAGCCTCGTGCTGTCGGACC	CCTTTGAGGCCCAAGGCCACA
*LIPA*	TCAAGGCTGCACCATAGGTTT	TTGAGAGACAACACGGGAGC
*RTN2*	TAGTGGAAGACCTGGTGGATT	GATGTGGCTCAACTGATTGG
*SMARCA4*	TGAGAACGCCAAGCAAGATG	GACACCAGCCACTCCAAACC
*XPO6*	GGAACTCGATGAGAGTTACATTG	ACCGGGAACTGGGAATAGGA
*PECAM1*	TCACAGATAAGCCCACCAGAG	ACAGAGCACCGAAGTACCATT
*CFDP1*	ACCAAAGTCAAAAGCAGCCC	TGACAAGAGCCTGCGGTTTT
*FES*	CGCCTTCGTGCAGACAATAC	GTGAGAAAGTCGCCCCCTTG
*HP*	TGGAGCACTTGGTTCGCTAT	CCCATTGCTTCTCGTCGTTT
*ZNF257*	GGAGCAAGGAAAAGAGCCCT	TGGGCAAAGGTCTTCAGCAA
*GAPDH*	ACTCTTCCACCTTCGATGCC	TGGGATAGGGCCTCTCTTGC

### 2.14 Western blot

After weighing and homogenizing the samples, they were subjected to centrifugation, and the protein concentration in the resulting supernatant was measured. The protein expression levels of LIPA, RTN2, PECAM1, CFDP1, XPO6, and GAPDH were assessed using antibodies. These antibodies included anti-LIPA (1:1000, Proteintech Group, Wuhan, China), anti-RTN2 (1:1000, Proteintech Group, Wuhan, China), anti-PECAM1 (1:2000, Proteintech Group, Wuhan, China), anti-CFDP1 (1:500, Proteintech Group, Wuhan, China), anti-XPO6 (1:1000, Proteintech Group, Wuhan, China), anti-iNOS (1:1000, selleck, Houston, The United States), anti-IL-6 (1:1000, Proteintech Group, Wuhan, China), anti-IL-1β (1:2000, Proteintech Group, Wuhan, China), and anti-GAPDH (1:3000, Proteintech Group, Wuhan, China). Western blot band intensities were quantified using ImageJ software (National Institutes of Health, USA). For each blot, the same exposure conditions were applied to all samples to avoid signal saturation. Raw images were converted to 8-bit grayscale, and the integrated density of each band was measured after background subtraction using a rolling ball algorithm. Target protein expression levels were normalized to the corresponding internal loading control (GAPDH) from the same lane to account for variations in protein loading and transfer efficiency. Normalized values were then expressed relative to the mean value of the control group, which was set to 1. The final data are presented as mean ± standard error of the mean (SEM) and were subjected to statistical analysis as described in the Statistical Analysis section.

### 2.15 Immunohistochemistry

Paraffin-embedded heart sections (4 um thick) were deparaffinized in xylene and rehydrated through a graded series of ethanol. Antigen retrieval was performed by immersing the sections in 0.01 M sodium citrate buffer (pH 6.0) and heating in a microwave at medium power until boiling, followed by an 8-minute stand and a final 7-minute incubation at low power. After cooling to room temperature, the sections were rinsed three times with phosphate-buffered saline (PBS, pH 7.4). To quench endogenous peroxidase activity, sections were incubated in 3% H_2_O_2_ in the dark for 25 minutes, followed by three PBS washes on an orbital shaker (100 rpm). Non-specific binding was blocked with 3% bovine serum albumin (BSA) for 30 minutes at room temperature.

The sections were then incubated overnight at 4 °C in a humidified chamber with primary antibodies against RTN2 (1:200), LIPA (1:2000), PECAM1 (1:2000), XPO6 (1:100), CFDP1 (1:200), Ly6G (1:200) and Ly6C (1:500). After washing three times with PBS, the sections were incubated with horseradish peroxidase (HRP)-conjugated secondary antibodies for 50 minutes at room temperature. The signal was visualized using a 3,3’-diaminobenzidine (DAB) substrate kit, followed by counterstaining with hematoxylin for 3 minutes. After differentiation with hydrochloric acid-ethanol and dehydration, the slides were mounted and examined using a light microscope.

### 2.16 Culture and stimulation of RAW264.7 cells

The mouse monocyte-macrophage line RAW264.7, purchased from the Servicebio (Wuhan, China), was maintained in (DMEM)/F12 supplemented with 10% FBS containing 10% FBS, 100 U/mL penicillin, and 100 mg/mL streptomycin at 37 °C and under 5% CO2. RAW264.7 cells were transfected with LIPA siRNAs. The serum-free medium, siRNA, and LipoRNAi transfection reagent were mixed directly, incubated at room temperature for 20 min, and then were directly added to the cell culture vessel. The transfected cells were incubated at 37 °C and 5% CO_2_ for 48 h. The RAW264.7 cells were treated with LPS (100 ng/ml) and IFN-γ (10 ng/ml) to induce a proinflammatory phenotype, and the control group was treated with PBS. After stimulation for 12 h, the cultured cells were used for further analysis.

### 2.17 Data analysis

Multiple testing correction includes TWAS, differential expression analysis, functional enrichment analysis (GO/KEGG), and correlation matrix analysis. Experimental data are presented as mean ± SD. Statistic analysis on data in RT-qPCR and Western blot were evaluated for homoscedasticity and normality assumption. For the comparison of 2 groups, the unpaired 2-tailed Student t test was used, followed by the Mann-Whitney correction for nonparametric data. The statistical analysis was performed using IBM SPSS Statistics version 25 software, and P<0.05 were considered statistically significant.

## 3 Result

### 3.1 TWAS revealed the association between blood gene expression and AMI risk

**Figure 2:**
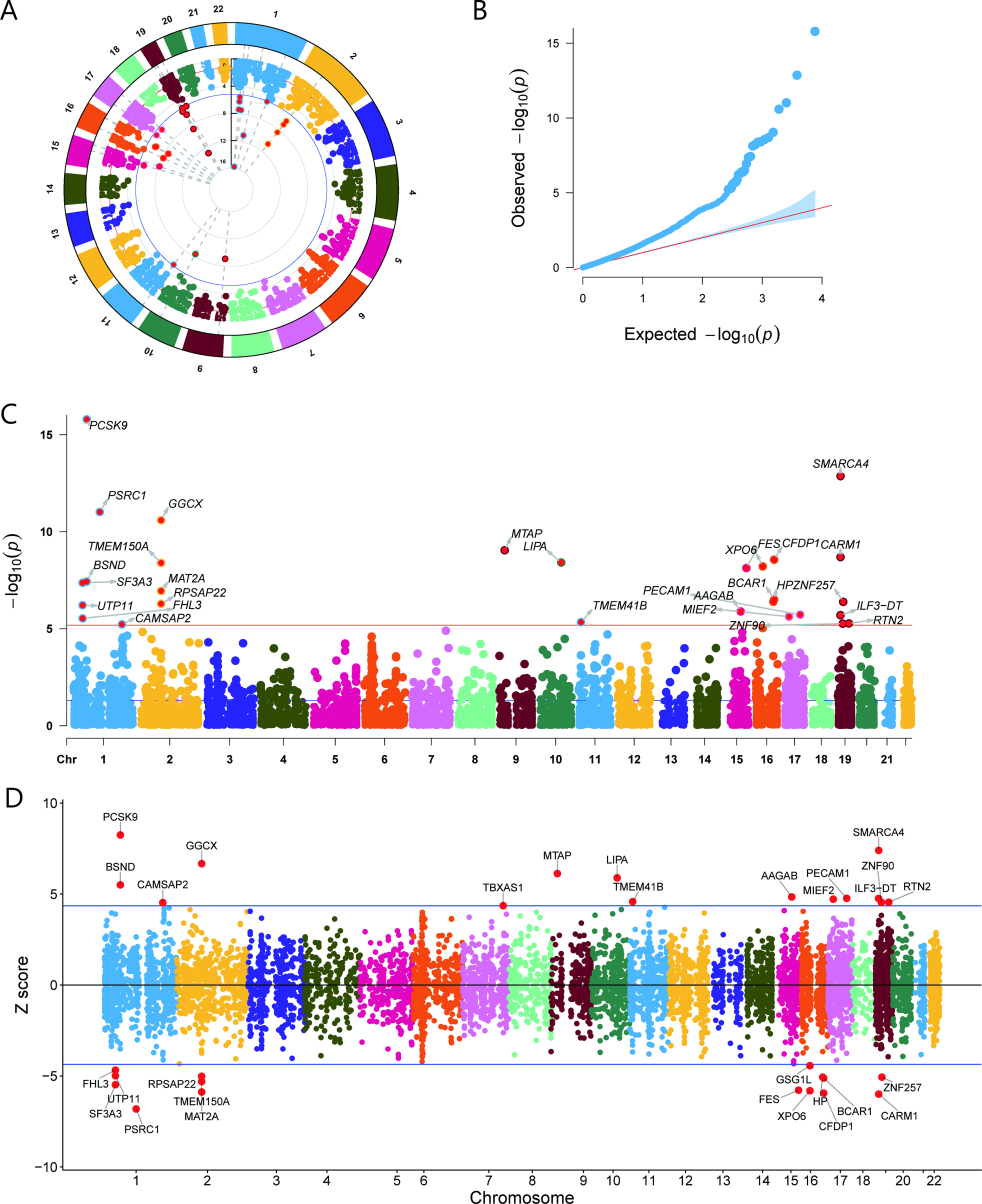
TWAS was used to analyze the association between blood gene expression and AMI risk. **(A)** Circular Manhattan plot showing significant AMI-related genes on chromosomes. **(B)** Quantile-Quantile plot for GWAS. **(C)** Rectangular Manhattan plot depicting significant AMI-related genes on chromosomes. **(D)** Z-score maps highlighting genes significantly associated with AMI, illustrating the effect size and direction of each genetic variant.

We used TWAS to assess the association between blood gene expression and the risk of AMI. TWAS identified 27 genes significantly associated with AMI risk (**Figures 2A** and **1B**). Specifically, each gene is marked by a specific SNP and chromosomal location. The results are represented by TWAS Z values (indicate the direction and magnitude of gene expression’s effect on AMI risk) and TWAS P values (indicate the significance of the association between gene expression and AMI risk). High expression levels of 13 genes, including *MTAP* (TWAS Z = 6.13, TWAS P = 9.04E-10) and *LIPA* (TWAS Z = 5.89, TWAS P = 3.88E-09), were associated with an increased risk of AMI. In contrast, high expression of 14 genes, including *BCAR1* (TWAS Z = -5.11, TWAS P = 3.14E-07) and *PSRC1* (TWAS Z = -6.81, TWAS P = 9.62E-12), was associated with a reduced risk of AMI (**Supplementary Table 1**) (**Figures 2C, D**).

### 3.2 GO and KEGG enrichment analyses

**Figure 3:**
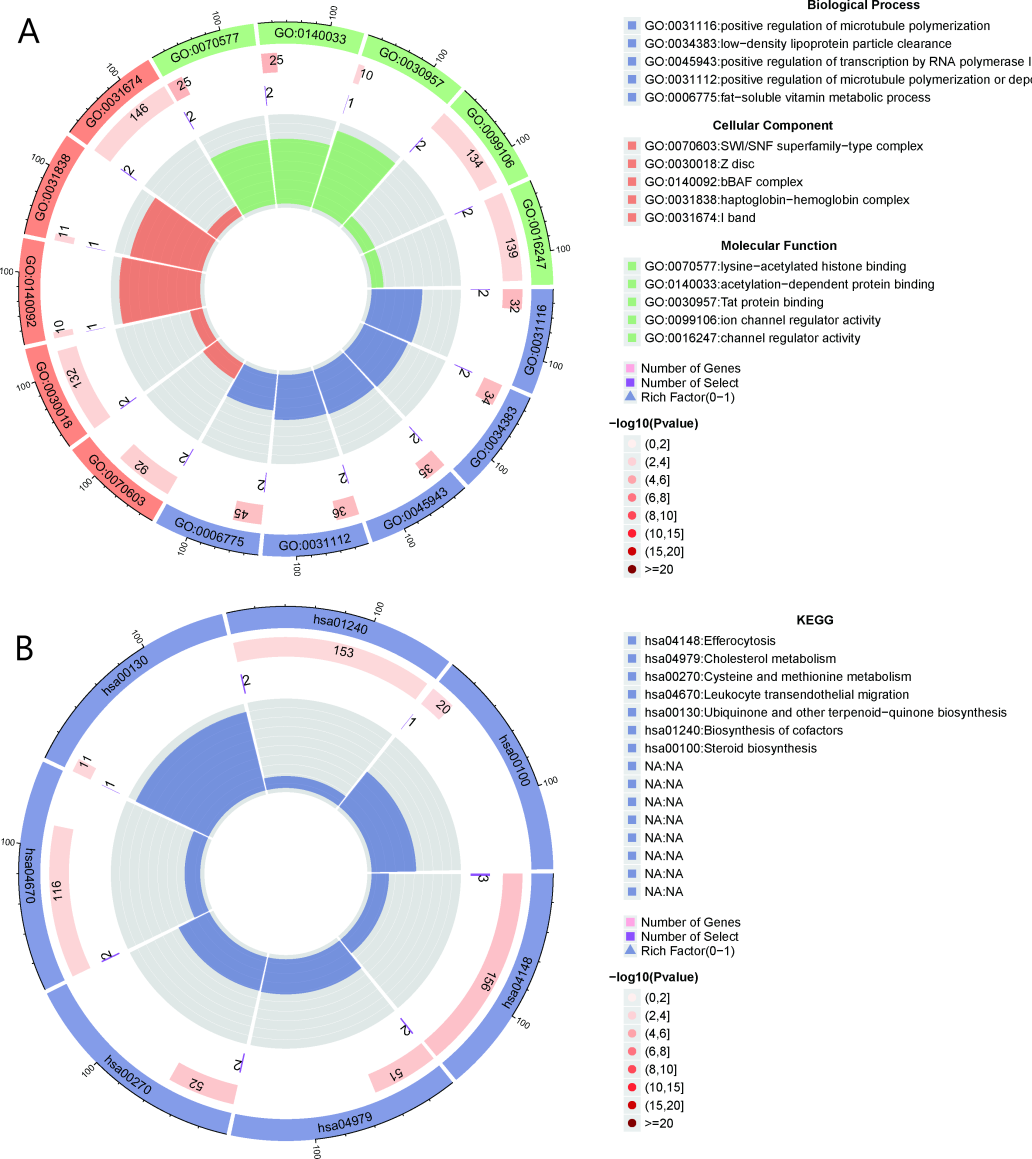
GO and KEGG enrichment analyses **(A)** The GO analysis for the AMI-related genes. **(B)** The KEGG analysis for the AMI-related genes.

GO analysis of the 27 AMI-related genes identified multiple biological processes, cellular components, and molecular functions associated with AMI (**Figures 3A**). In terms of biological processes, the *PSRC1* and *FES* genes are involved in the positive regulation of microtubule polymerization, while *LIPA* and *PCSK9* are associated with low-density lipoprotein particle clearance. *SMARCA4* and *CARM1* are involved in the positive transcriptional regulation of RNA polymerase I. Fat-soluble vitamin metabolism is also linked to the *LIPA* and *GGCX* genes. In the context of cellular components, *SMARCA4* and *CFDP1* are associated with SWI/SNF superfamily complexes, whereas *RTN2* and *FHL3* are linked to the Z disc and I band. Additionally, *SMARCA4* is associated with the bBAF complex and Tat protein binding. Regarding molecular functions, the *SMARCA4* and *CARM1* genes are closely related to lysine acetylated histone binding and acetylation-dependent protein binding. In contrast, *BSND* and *PCSK9* are associated with ion channel regulatory activity.

KEGG analysis further provided insights into the involvement of these genes in various biological processes. In the category of cellular processes, *BCAR1*, *LIPA*, and *PECAM1* are implicated in efferocytosis, suggesting their role in cell transport and catabolism. In the biological systems category, *LIPA* and *PCSK9* are linked to cholesterol metabolism, while *BCAR1* and *PECAM1* are associated with the transendothelial migration of white blood cells, indicating their involvement in immune system function. In terms of metabolism, *MTAP* and *MAT2A* are involved in cysteine and methionine metabolism, while *GGCX* plays a role in the biosynthesis of ubiquinone and other terpenoid quinones, contributing to the cofactor synthesis pathway. Additionally, *LIPA* is associated with steroid biosynthesis, highlighting its importance in lipid metabolism (**Figure 3B**).

### 3.3 Construction and testing of the AMI risk prediction model

**Figure 4:**
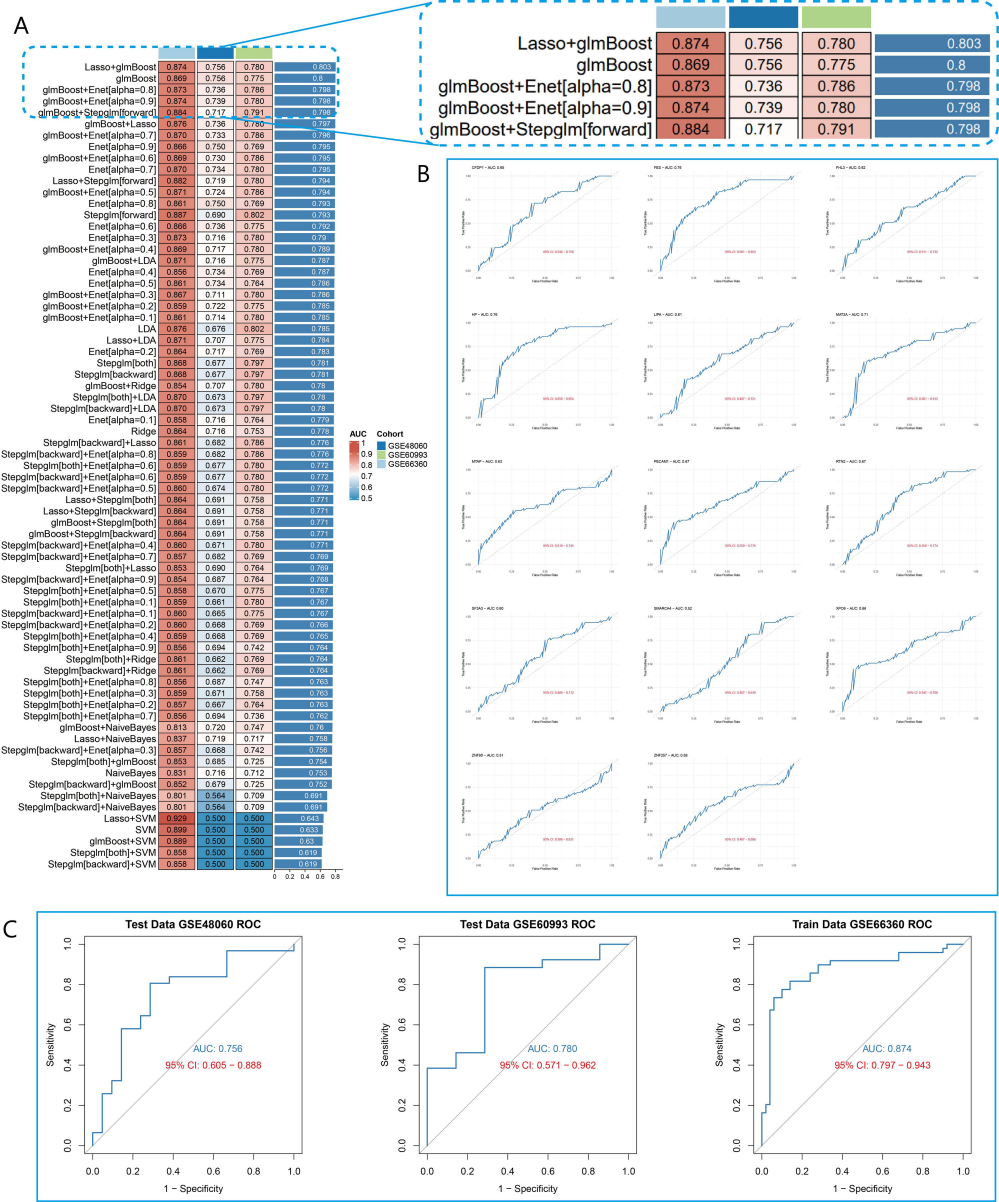
Construction and testing of the AMI risk prediction model. **(A)** The optimal prediction model, Lasso+glmBoost algorithm, was selected based on the AUC values obtained from 12 machine learning algorithms and 101 parameter combinations. **(B)** ROC curve analysis of 14 genes identified in the Lasso+glmBoost algorithm. **(C)** ROC curves were used in the optimal prediction model to identify patients with AMI and controls with accuracy in the GSE66360, GSE48060, and GSE60993 cohorts.

We integrated 12 machine learning algorithms to develop a comprehensive risk prediction model for AMI. The GSE66360 dataset was used as the training set, while the GSE48060 and GSE60993 datasets were used as the validation sets. The average area under the curve (AUC) of the 12 machine learning models was calculated across the different cohorts. Notably, five models exhibited high average accuracy. And the Lasso+glmBoost algorithm demonstrated the best performance (**Figure 4A**).

To further assess model performance, we employed ROC curve analysis. The Lasso+glmBoost model achieved an AUC of 0.874 (95% CI: 0.797–0.943) in the training set (GSE66360), an AUC of 0.756 (95% CI: 0.605–0.888) in the validation set GSE48060, and an AUC of 0.780 (95% CI: 0.571–0.962) in the validation set GSE60993 (**Figure 4C**). Additionally, in the Lasso+glmBoost model, genes such as *MTAP, LIPA, RTN2, SMARCA4, CFDP1, MAT2A, XPO6, FES, FHL3, SF3A3, ZNF257, ZNF90, HP*, and *PECAM1* all demonstrated strong diagnostic performance (**Figure 4B**). These findings suggest that these genes can effectively identify patients at risk for AMI and may provide valuable insights for optimizing clinical decision-making in AMI management.

### 3.4 Bayesian colocalization analysis

Bayesian colocalization analysis revealed that nine key genes (*LIPA, PECAM1, SMARCA4, HP, RTN2, CFDP1, XPO6, FES*, and *ZNF257*)showed significant colocalization with AMI. These results suggest a common causal genetic driver linking the expression of these genes to AMI (PPH4 > 0.8). Among these genes, *LIPA* (PP4 = 0.996), *PECAM1* (PP4 = 0.991), *SMARCA4* (PP4 = 0.984), HP (PP4 = 0.966), *RTN2* (PP4 = 0.962), *CFDP1* (PP4 = 0.948), *XPO6* (PP4 = 0.947), *ZNF257* (PP4 = 0.945), and FES (PP4 = 0.847) exhibited high colocalization probabilities, suggesting that they are likely involved in AMI through shared genetic mechanisms. In contrast, *ZNF90* (PP4 = 0.105) showed moderate colocalization probabilities, while *FHL3* and *SF3A3* (both PP4 = 0.077) exhibited low colocalization probabilities, implying that these genes may exert their effects through distinct pathways. Furthermore, although *MAT2A* (PP4 = 0.003) and *MTAP* (PP4 = 0.002) displayed lower colocalization probabilities, their higher PP3 values suggest that phenotypic-specific genetic variations may also contribute to their effects (**Table 3**) (**Supplementary Figure 2**).

**Table 3:** Bayesian colocalization analysis of 14 genes.

ID	COLOC.PP0	COLOC.PP1	COLOC.PP2	COLOC.PP3	COLOC.PP4
LIPA	0	0	0	0.004	0.996
PECAM1	0	0.004	0	0.005	0.991
SMARCA4	0	0	0.004	0.012	0.984
HP	0	0.024	0	0.01	0.966
RTN2	0.006	0.029	0.001	0.002	0.962
CFDP1	0	0	0	0.052	0.948
XPO6	0	0	0	0.053	0.947
ZNF257	0	0.017	0	0.038	0.945
FES	0	0.003	0	0.15	0.847
ZNF90	0	0.429	0	0.466	0.105
FHL3	0	0.103	0	0.82	0.077
SF3A3	0	0.103	0	0.82	0.077
MAT2A	0	0	0	0.997	0.003
MTAP	0	0	0.053	0.945	0.002

### 3.5 Immunoinfiltration analysis

**Figure 5:**
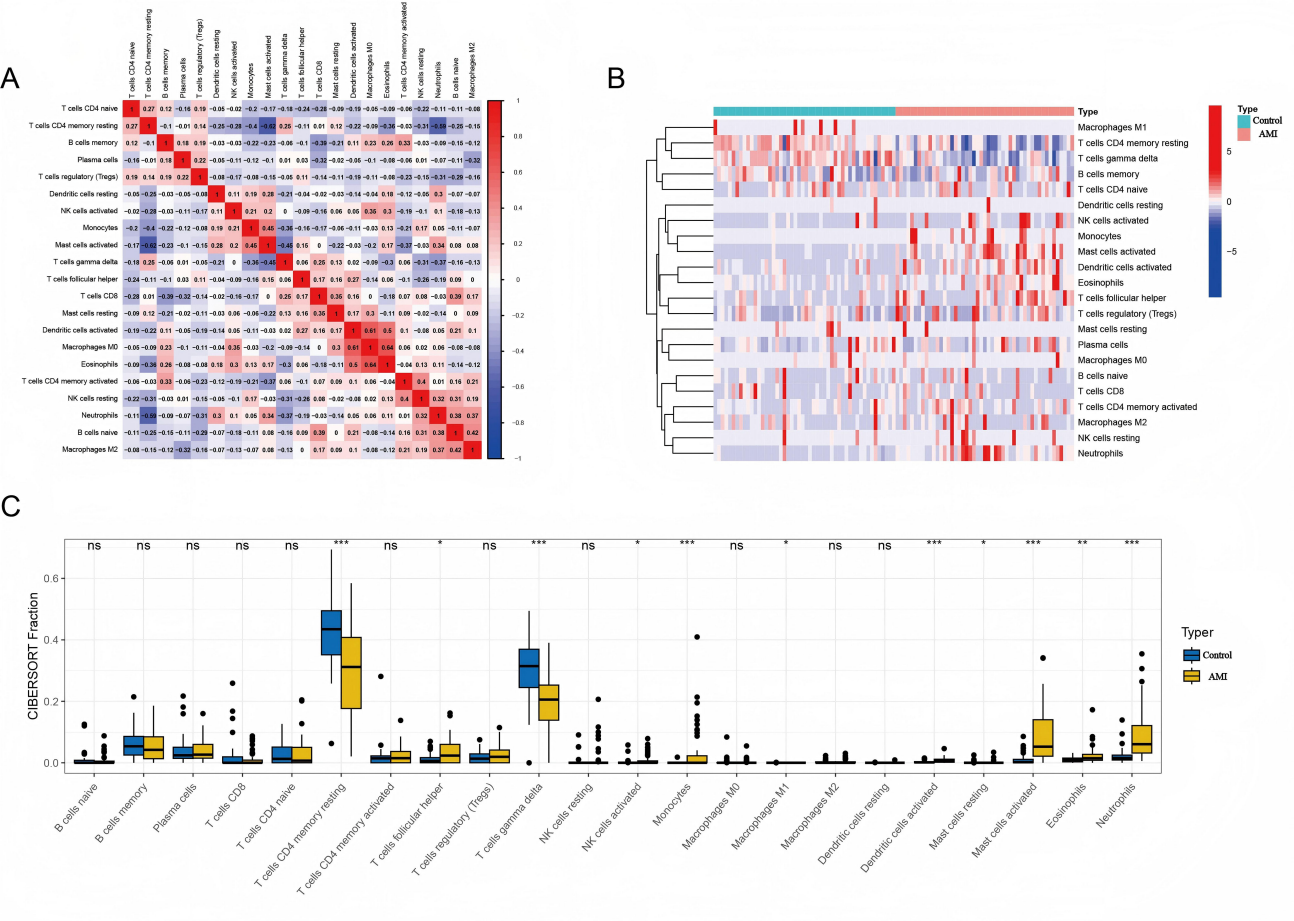
Distribution of infiltrating immune cells. **(A)** A correlation heatmap of the infiltrating immune cells. **(B)** Distribution of 22 immune cells in AMI and control groups. **(C)** Comparison of 22 immune cells between AMI and control groups. *p < 0.05, **p < 0.01, ***p < 0.001.

We used the CIBERSORT algorithm to analyze the proportion of 22 immune cell subsets in both AMI and control samples. The immune infiltration analysis revealed significant differences in the distribution of various immune cell types between the AMI and control groups (**Figure 5B**). Specifically, the infiltration levels of T follicular helper cells, activated NK cells, monocytes, activated dendritic cells, resting/activated mast cells, eosinophils, and neutrophils were significantly higher in the AMI group compared to the control group. In contrast, the levels of memory resting CD4^+^ T cells and γδ T cells were significantly lower in the AMI group than in the control group (**Figure 5C**). Additionally, potential interactions among the different immune cell types in AMI are depicted in **Figure 5A**. Consistently, IHC results demonstrated a significant increase in the infiltration of Ly6G neutrophils and Ly6C monocytes in the myocardium of the AMI group compared to the control group (**Supplementary Figure 3**).

### 3.6 Immunocorrelation analysis

**Figure 6:**
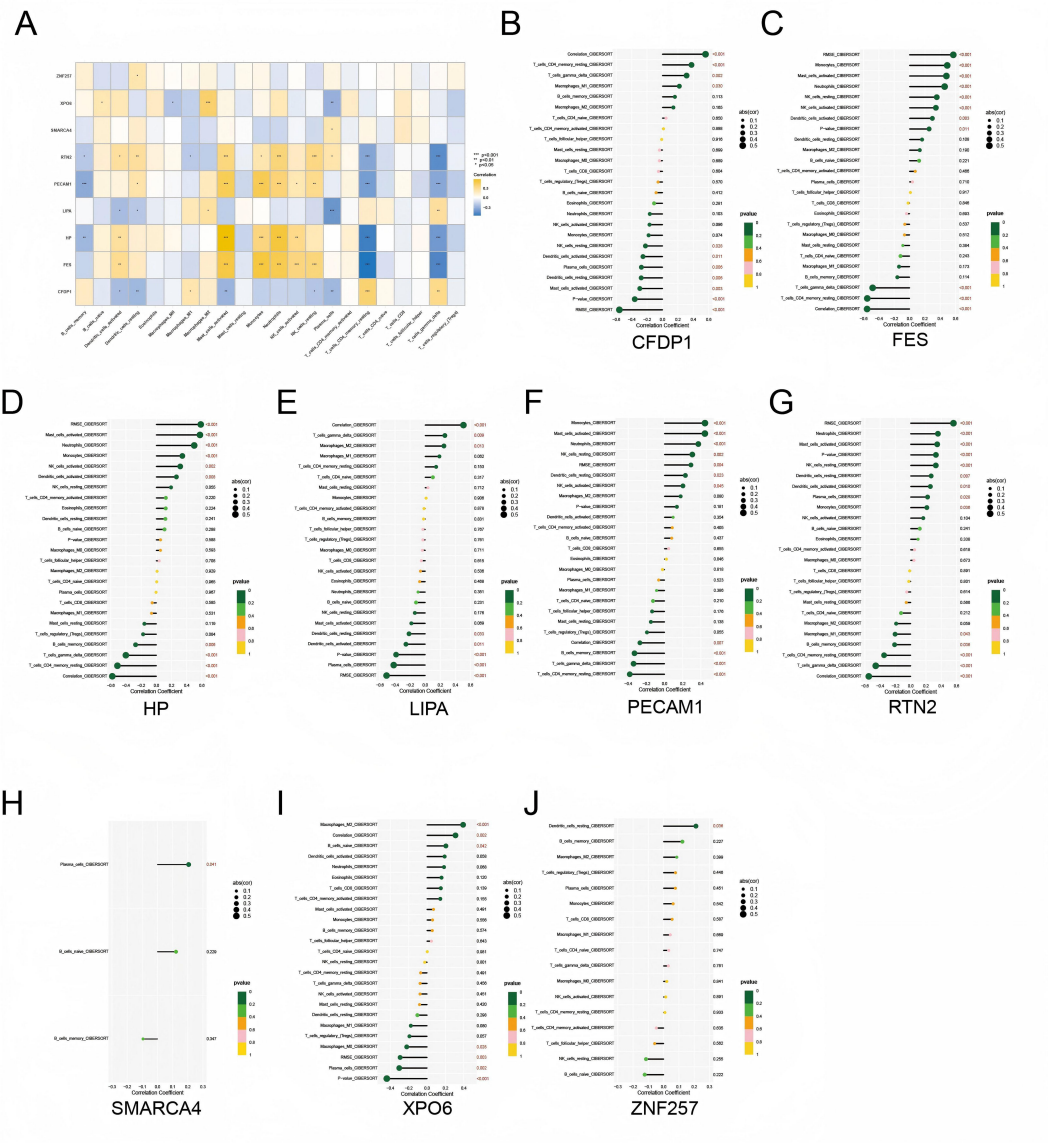
Correlation between nine key genes and infiltrating immune cells. **(A)** Correlation heat map between nine key genes and infiltrating immune cells. **(B–J)** Correlation analysis of CFDP1, FES, HP, LIPA, PECAM1, RTN2, SMARCA4, XPO6, and ZNF257 with various infiltrating immune cell types. *: P < 0.05, **: P < 0.01 and ***: P < 0.001.

This study also explored the association between nine key genes (*LIPA, PECAM1, SMARCA4, HP, RTN2, CFDP1, XPO6, ZNF257, FES*) and infiltrating immune cells to reveal their potential role in regulating immune cell function (**Figure 6A**). Specifically, *PECAM1* exhibited significant associations with a variety of immune cells, including memory B cells (r = -0.3311, p = 0.0008), γδT cells (r = -0.3412, p = 0.0005), resting natural killer cells (r = 0.3055, p = 0.0021), and neutrophils (r = 0.3716, p = 0.0002). *HP* was significantly associated with multiple immune cell types, including resting CD4+ memory T cells (r = -0.5130, p = 8.93E-08), γδT cells (r = -0.4018, p = 3.74E-05), activated mast cells (r = 0.5742, p = 5.16E-10), and neutrophils (r = 0.4986, p = 2.25E-07). *RTN2* showed significant associations with multiple immune cells, particularly resting dendritic cells (r = 0.2678, p = 0.0074) and neutrophils (r = 0.3565, p = 0.0003). *CFDP1* exhibited significant negative correlations with immune cells such as activated mast cells (r = -0.2947, p = 0.0031) and resting dendritic cells (r = -0.2764, p = 0.0056). *XPO6* did not show significant associations with most immune cell types. However, it was positively correlated with M2 macrophages (r = 0.39, p = 0.000059) and negatively correlated with M0 macrophages (r = -0.22, p = 0.028). *FES* was significantly associated with a variety of immune cells, including γδT cells (r = -0.4821, p = 4.35E-07), resting natural killer cells (r = 0.3543, p = 0.0003), activated mast cells (r = 0.4800, p = 4.97E-07), and neutrophils (r = 0.4571, p = 2.59E-06). *LIPA, SMARCA4* and *ZNF257* did not show significant associations with most immune cell types (**Figures 6B–J**).

### 3.7 Gene set enrichment analysis of key genes

**Figure 7:**
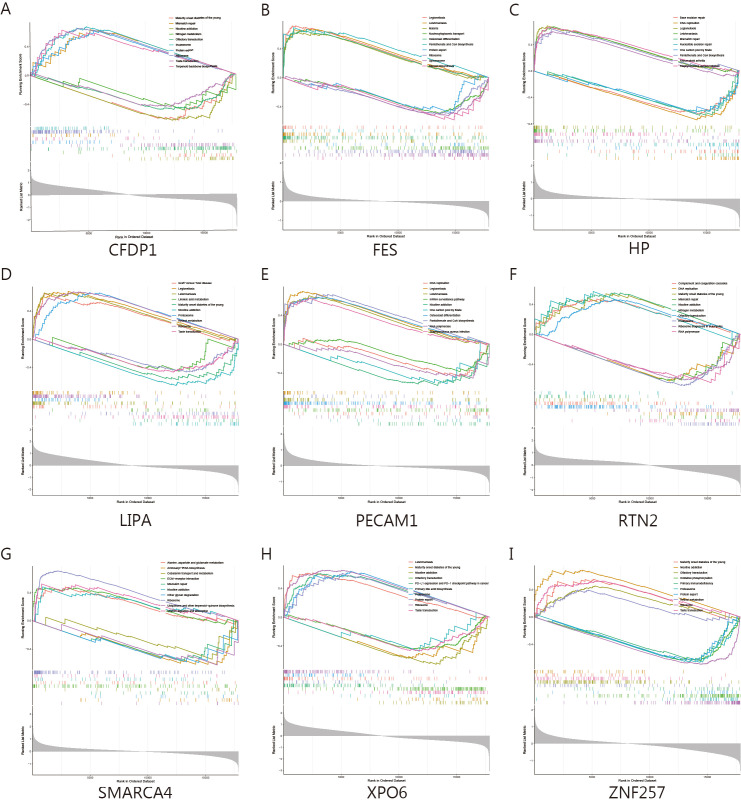
GSEA analysis of key genes for AMI. **(A–I)** Single gene GSEA analysis of LIPA, PECAM1, SMARCA4, HP, RTN2, CFDP1, XPO6, ZNF257, and FES revealed the top 5 upregulated and 5 downregulated pathways for each gene.

Single-gene GSEA revealed that *CFDP1* is associated with the upregulation of biological processes such as mismatch repair and protein synthesis. *FES*, on the other hand, is closely linked to processes like protein synthesis, steroid biosynthesis, and cytoplasmic transport. Similarly, *XPO6* is strongly associated with the upregulation of *PD1, PDL1*, and protein synthesis. *HP* and *PECAM1*, both upregulated in diseases such as Legionella and Staphylococcus aureus infections, are also implicated in the downregulation of DNA replication and base excision repair processes. *RTN2* and *SMARCA4* are closely associated with the upregulation of nitrogen metabolism, as well as alanine, aspartate, and glutamate metabolism. Lastly, *CFDP1, LIPA*, and *ZNF257* are strongly linked to Maturity onset diabetes of the young (**Figures 7A–I**).

### 3.8 Gene set variation analysis of key genes

**Figure 8:**
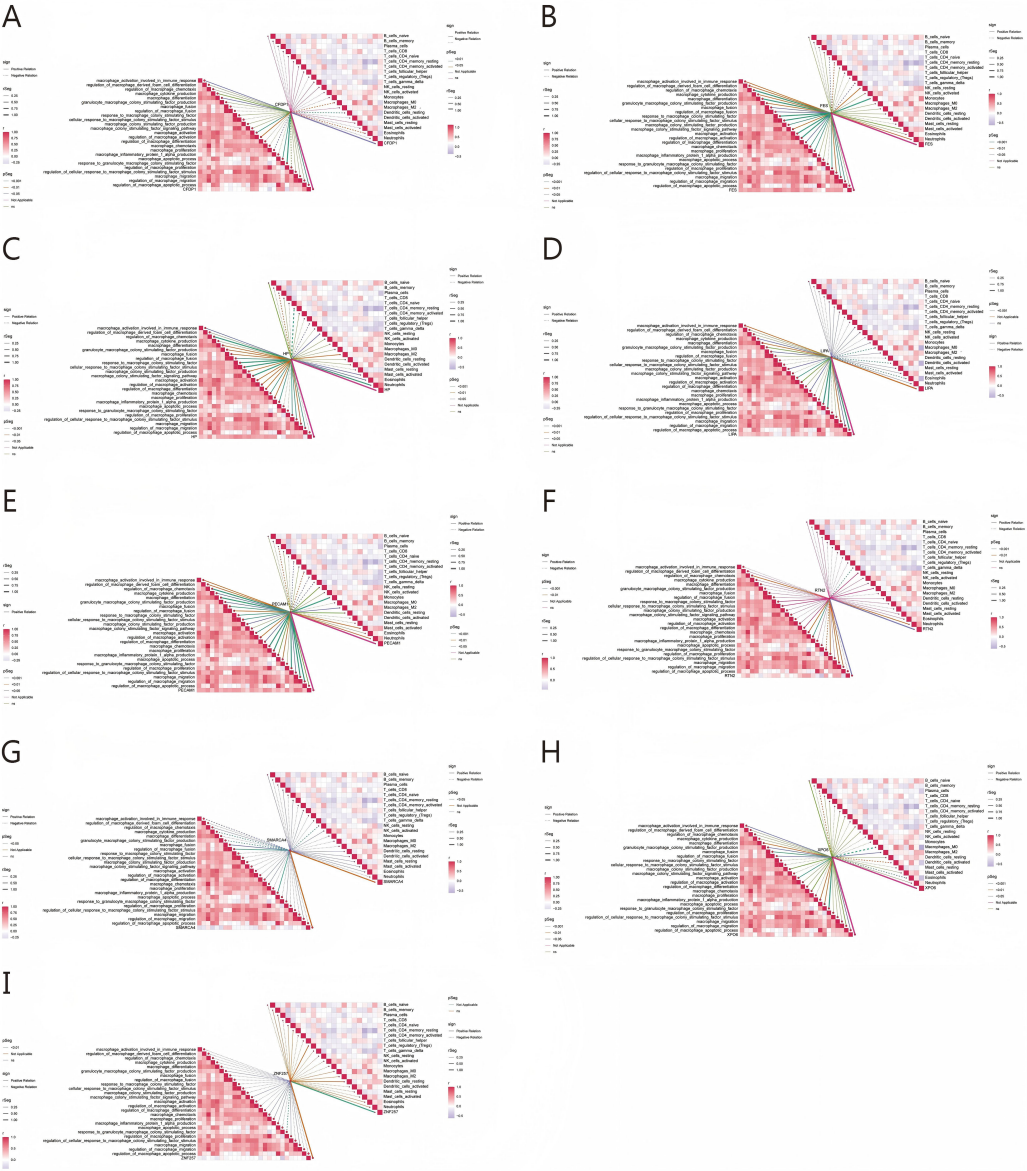
GSVA analysis of key genes for AMI. **(A–I)** Correlation Analysis of LIPA, PECAM1, SMARCA4, HP, RTN2, CFDP1, XPO6, ZNF257, FES with immune cells and macrophage related biological processes.

We further employed GSVA analysis to explore the correlation of nine key genes with macrophage regulation related biological processes and different immune cells. *CFDP1* was positively correlated with macrophage apoptosis. *XPO6* showed a negative correlation with M0 macrophages and a positive correlation with M2 macrophages. *HP* and *PECAM1* were strongly associated with Tregs and resting memory CD4+ T cells. *LIPA* and *PECAM1* were positively correlated with macrophage activation, differentiation, proliferation, and cytokine production. *SMARCA4* was negatively correlated with resting NK cells but positively correlated with the production of macrophage inflammatory protein 1α. *RTN2* was involved in macrophage proliferation, chemotaxis, and migration. In contrast, *ZNF257* showed no significant association with biological processes related to the regulation of immune cells and macrophages (**Figures 8A–I**).

### 3.9 Single-cell RNA sequencing analysis of key genes

To elucidate the cell-type-specific expression profiles of these candidate genes following AMI, we analyzed publicly available cardiac single-cell RNA sequencing datasets. Our results demonstrated that PECAM1, LIPA, and CFDP1 were significantly upregulated in several key populations, including endothelial cells, monocytes, and M1 macrophages. Notably, PECAM1 exhibited a high expression frequency and robust average expression levels within NK cells. LIPA was markedly enriched in the monocyte/macrophage lineage, suggesting that its activation may drive immune-mediated inflammation within the infarcted territory. Furthermore, the stable expression of CFDP1 in tissue stem cells indicates its potential involvement in post-AMI tissue regeneration or cytoprotective responses against cellular stress (**Supplementary Figure 1**).

### 3.10 Gene expression verification

**Figure 9:**
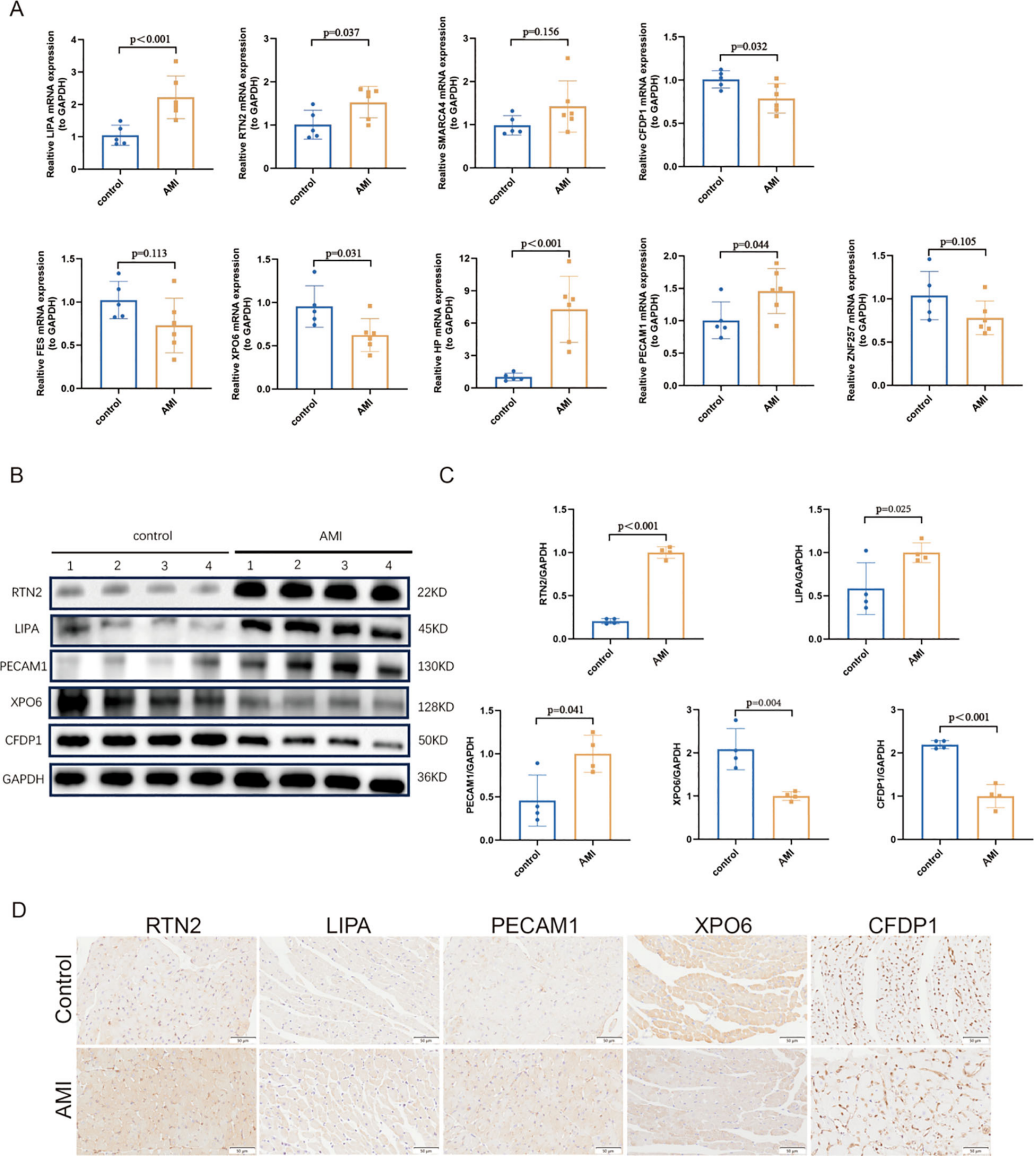
**(A)** Verification of the nine key genes by RT-qPCR (AMI samples = 6, control samples =5). **(B)** and **(C)**. The protein expression levels of LIPA, RTN2, PECAM1, CFDP1 and XPO6 in the heart were analyzed by Western blot. **(D)** The immunohistochemistry representative graph of LIPA, RTN2, PECAM1, CFDP1 and XPO6 in the heart. *: P < 0.05, **: P < 0.01 and ***: P < 0.001.

To further investigate the expression pattern of key genes, RT-qPCR and Western blot was performed on left ventricular tissue from both the control and AMI groups. The results revealed that, compared to the control group, the expression levels of *LIPA, RTN2*, and *PECAM1* were significantly increased in the AMI group. Moreover, the RT-qPCR indicated that the expression of *HP* was significantly increased in AMI. In contrast, the expression levels of *CFDP1* and *XPO6* were notably decreased. No statistically significant difference was observed in the expression of *ZNF257, SMARCA4* and *FES* between the two groups. This lack of difference may be attributed to the disparity in sample sizes between the experimental and dataset samples (**Figure 9**).

Furthermore, We performed an RT-qPCR time-course analysis of Lipa expression in heart tissues at 0h, 24h, 48h, and 1 week post-MI. The result revealed that *LIPA* mRNA expression began to significantly increase at 24h post-MI, reached its peak at 48h, and maintained a sustained high expression level compared to the pre-MI baseline even at 1 week post-MI. To further investigate the functional role of LIPA in macrophages, an inflammatory (M1-like) phenotype was induced by stimulation with LPS and IFN-γ. It was demonstrated that siRNA-mediated knockdown of LIPA significantly suppressed the expression of key pro-inflammatory cytokines, including IL-1β, IL-6, and iNOS, at both the mRNA and protein levels (**Supplementary Figure 4**).

## 4 Discussion

AMI is one of the leading cardiovascular diseases globally, often resulting in major adverse cardiovascular events. Early diagnosis and treatment are critical in improving patient outcomes and reducing mortality rates (20). Increasing evidence suggests that genetic factors play a pivotal role in the onset and progression of AMI. Concurrently, with advancements in bioinformatics, machine learning algorithms have become increasingly sophisticated and are now widely utilized for predicting disease biomarkers and therapeutic targets (8). In this study, we identified nine key genes associated with AMI through the integration of TWAS, 12 machine learning methods, and Bayesian colocalization analysis. ROC curve analysis demonstrated that these nine key genes exhibited excellent diagnostic efficiency. Additionally, we employed various bioinformatics tools, including enrichment analysis, immunoinfiltration analysis, Single-gene GSEA, and GSVA, to explore the molecular mechanisms underlying the role of these genes in AMI. Finally, we validated the expression levels of these nine key genes in a mouse model of myocardial infarction by qRT-PCR and western blot. Our findings provide novel targets for the diagnosis and treatment of AMI and contribute to a deeper understanding of its pathogenesis.

First, we identified 27 significant genes associated with AMI by TWAS. Functional enrichment analyses indicated that the AMI-related genes identified in this study were mainly involved in several biological pathways closely associated with the pathogenesis of acute myocardial infarction, including lipid metabolism, leukocyte transendothelial migration, immune and inflammatory responses, and macrophage activation. These biological processes represent key mechanisms underlying coronary atherosclerosis, plaque instability, and post-infarction inflammatory remodeling. Among these pathways, lipid metabolism plays a central role in the development of coronary artery disease and AMI. Dysregulation of cholesterol metabolism and impaired clearance of low-density lipoprotein (LDL) particles can promote lipid accumulation within arterial walls and accelerate atherosclerotic plaque formation. Consistent with this mechanism, genes such as LIPA and PCSK9 identified in our enrichment analysis were associated with cholesterol metabolism and LDL particle clearance. Previous studies have shown that early reduction of LDL levels following AMI significantly decreases cardiovascular risk and adverse clinical outcomes (21, 22). These findings support the biological relevance of the lipid-metabolism–related pathways identified in our analysis. In addition to metabolic pathways, immune and inflammatory processes are critical drivers of myocardial injury and tissue remodeling following infarction (23). The enrichment of pathways related to leukocyte transendothelial migration suggests that several identified genes may contribute to the recruitment of immune cells into ischemic myocardial tissue. After coronary occlusion, neutrophils and monocytes rapidly infiltrate the infarcted myocardium, where they release inflammatory mediators and contribute to tissue damage as well as subsequent repair processes. Our immunoinfiltration analysis further supported this concept, showing significant correlations between several key genes and immune cell subsets, including neutrophils, macrophages, and dendritic cells. Furthermore, GSVA analysis highlighted strong associations between several key genes and macrophage-related biological processes, including macrophage activation, differentiation, proliferation, and cytokine production. Macrophages play a central role in both the inflammatory and reparative phases of myocardial infarction. During the early phase of AMI, pro-inflammatory macrophages contribute to the clearance of necrotic tissue and amplify inflammatory signaling, whereas reparative macrophages promote tissue repair and cardiac remodeling (24). The observed correlations between genes such as LIPA, PECAM1, and RTN2 and macrophage regulatory processes suggest that these genes may influence AMI progression through modulation of macrophage-mediated inflammatory responses. Although some enriched pathways were annotated in infection-related categories, these pathways likely reflect shared inflammatory and innate immune signaling mechanisms that are also activated during myocardial infarction. Taken together, the enrichment, GSEA, and GSVA analyses consistently indicate that metabolic dysregulation and immune-mediated inflammation are key mechanisms through which the identified genes may contribute to AMI pathogenesis.

Based on bioinformatics approaches, including machine learning and Bayesian colocalization analysis, we identified nine key genes and conducted functional analysis to further investigate their roles in AMI. Several genes identified in this study, including LIPA, PECAM1, FES, and HP, have previously been implicated in cardiovascular diseases such as coronary artery disease and myocardial infarction. Our results therefore provide additional validation and mechanistic insights into their roles in lipid metabolism and inflammatory responses in AMI. In addition, SMARCA4 has been reported to participate in cardiac stress responses and myocardial injury in experimental studies. Our analysis further supports its potential involvement in AMI and suggests that chromatin remodeling–related regulatory mechanisms may contribute to myocardial infarction pathogenesis. In contrast, several other genes identified in this study, including RTN2, CFDP1, XPO6, and ZNF257, have been rarely investigated in the context of myocardial infarction. These findings therefore suggest potentially novel molecular mechanisms involved in immune regulation and inflammatory responses during AMI.

*LIPA* is an enzyme that hydrolyzes cholesterol esters and triglycerides within cellular lysosomes to produce free cholesterol and fatty acids (25). Previous GWAS have shown that *LIPA* is strongly associated with the risk of atherosclerosis, coronary artery disease (CAD), and myocardial infarction (26). In this study, we observed that *LIPA* expression is elevated in the hearts of mice with myocardial infarction and demonstrated significant diagnostic potential. *LIPA* is primarily implicated in metabolic and infectious diseases, with a particular role in LDL particle clearance and lipid metabolism. Furthermore, immunoinfiltration and GSVA revealed that *LIPA* is positively correlated with the activation, differentiation, proliferation, and cytokine production of macrophages. Recent work in Ldlr^-^/^-^ mice fed a Western diet showed that overexpression of *LIPA* resulted in larger atherosclerotic lesions accompanied by altered macrophage function (27). These changes were attributed to *LIPA*-mediated disturbances in lipid metabolism and the enhanced release of inflammatory mediators, which in turn promoted pro-inflammatory polarization and functional reprogramming of macrophages (M1). Collectively, these findings suggest that upregulated *LIPA* may aggravate atherosclerosis progression and plaque instability, thereby potentially increasing susceptibility to AMI.

*RTN2* is a protein involved in the dynamic remodeling of the endoplasmic reticulum (28). It plays a critical role in maintaining the structure and function of the endoplasmic reticulum and has been associated with conditions such as motor neuropathy, and cancer (29, 30). In this study, we observed that *RTN2* expression was elevated in the hearts of mice with AMI and was significantly associated with an increased risk of AMI. Enrichment analysis indicated that *RTN2* was linked to Z-disc and I band, potentially reflecting its role in regulating Ca^2+^ efflux from the endoplasmic reticulum. GSEA further suggested that *RTN2* was upregulated in processes related to the complement and coagulation cascade, as well as in metabolic diseases. Immunoinfiltration analysis revealed a significant correlation between *RTN2* expression and various immune cell types, including resting dendritic cells and neutrophils. Additionally, GSVA demonstrated that *RTN2* was positively correlated with macrophage proliferation, chemotaxis, and migration. A study by Song et al. demonstrated that *RTN2* mediates the proliferation and migration of gastric cancer cells by activating the ERK signaling pathway, which is correlated with by Ca2+ efflux from the endoplasmic reticulum (ER) (30). Taken together, *RTN2*, as a regulator of ER morphology and Ca²⁺ dynamics, may enhance neutrophil and macrophage migration and expansion via an ER–Ca^2+^–ERK axis, thereby amplifying chemokine and adhesion molecule expression and contributing to the inflammatory response in AMI.

*SMARCA4* (also known as *BRG1*) is the catalytic core of the SWI/SNF chromatin remodeling complex, playing a key role in epigenetic regulation through chromatin remodeling. Recent studies have shown that in a mouse model of AMI, *BRG1* expression is elevated in the myocardial infarction boundary region. Additionally, *BRG1* knockdown has been shown to improve ventricular arrhythmias following myocardial infarction in mice (31). However, other studies suggest that *BRG1* may exert a protective effect on the heart in myocardial infarction through pathways such as the *BRG1-NRF2-HO1* axis and the *BRG1/Nrf2/STAT3* pathway (32, 33). In our study, we found that *BRG1* shows a trend of increased expression in the hearts of mice with myocardial infarction. Enrichment analysis revealed that BRG1 was primarily involved in epigenetic regulation of RNA polymerase I transcription, amino acid acetylated histone binding, and acetylation-dependent protein binding. However, *SMARCA4* exhibited only a weak association with various immune cells. These findings suggest that the role of *SMARCA4* in myocardial infarction may be complex and multifaceted. Our study provides new insights into the mechanisms underlying the action of *SMARCA4* in myocardial infarction and lays the groundwork for future investigations into its potential therapeutic applications.

*CFDP1* is a protein-coding gene that belongs to the evolutionarily conserved Bucentaur family. Previous studies have shown that *CFDP1* is associated with aortic diameter, carotid media thickness, and CAD risk, and is essential for proper heart development (34, 35). Loss of *CFDP1* expression can lead to fatal Cardiac dysfunction (36). In our study, we found that *CFDP1* expression was downregulated in the heart of mice with AMI, and its expression was negatively correlated with immune cell types such as resting dendritic cells and activated mast cells. GSVA further revealed that *CFDP1* was positively correlated with macrophage apoptosis. To our knowledge, there are currently no studies directly examining the relationship between *CFDP1* and neutrophils or macrophages. Based on our data, we speculate that *CFDP1* may be involved in promoting apoptosis or turnover of specific immune cell populations, thereby contributing to the resolution of inflammation and potentially attenuating myocardial injury after AMI. This is worthy of further exploration in the future.

*XPO6* primarily mediates the export of proteins. Dysregulation of protein export has been implicated in a variety of diseases, including cancer and immune disorders (37). In our study, we observed a reduction in *XPO6* expression in the heart of mice with myocardial infarction. GSEA indicated that *XPO6* is associated with the upregulation of PD1 and PDL1 pathways. Immunoinfiltration and GSVA further revealed that *XPO6* expression was positively correlated with M2 macrophage abundance, suggesting a potential link to anti-inflammatory or reparative macrophage phenotypes in AMI. However, in chronic obstructive pulmonary disease, *XPO6* has been shown to promote the nuclear export of TLR2 mRNA in monocytes, thereby activating the MyD88/NF-κB signaling pathway and enhancing inflammatory responses (38). Therefore, *XPO6* may play a role in immune inflammation in acute myocardial infarction by regulating it.

*FES*, a member of the tyrosine kinase family, is involved in a variety of cellular processes, including cell motility, proliferation, differentiation, survival, and inflammation (39). our study revealed that *FES* shows a trend of decreased expression in the heart of mice with myocardial infarction. Moreover, *FES* is associated with several biological processes, including pantothenic acid and coenzyme A biosynthesis, nucleoplasmic transport, and protein export. It is also involved in regulating various immune cells, such as primitive CD4^+^ T cells and resting memory CD4^+^ T cells. Recent studies have shown that *FES* has protective effects on AMI and CAD (40). Inhibition or loss of *FES* promotes monocyte migration and accelerates atherosclerosis *in vivo*, whereas *FES* deficiency in lung cancer cells has been associated with mitochondrial dysfunction. Additionally, *FES* activity has been implicated in the regulation of glycemic control, blood pressure, and lipid metabolism (41, 42). In summary, *FES* may exert cardiovascular protection by modulating inflammatory and immune responses, mitochondrial function, and systemic metabolic homeostasis. Nevertheless, despite accumulating evidence for a beneficial role of *FES* in ischemic heart disease, the precise mechanisms by which *FES* influences the onset and progression of AMI remain to be fully elucidated.

*HP* is an acute phase α-glycoprotein that prevents oxidative tissue damage by removing free hemoglobin. Recent study has demonstrated that Haptoglobin exerts a protective effect in AMI (43). In our study, immunoinfiltration and GSVA revealed that *HP* expression was significantly negatively correlated with Tregs and resting CD4^+^ memory T cells, suggesting a potential role in modulating immune responses in these conditions.

*PECAM1* (also known as *CD31*) is primarily expressed in endothelial cells, platelets, white blood cells, and hematopoietic precursor cells. Several studies have demonstrated a significant positive correlation between *PECAM1* and myocardial infarction, with its elevated levels proposed as a potential biomarker for early detection (44, 45). Consistent with these findings, our study also observed elevated *PECAM1* expression in the heart of mice with myocardial infarction. Immunoinfiltration analysis showed that *PECAM1* expression was significantly correlated with neutrophils, memory B cells, γδT cells, and other immune cells. Additionally, enrichment analysis revealed that *PECAM1* is involved in apoptotic cell clearance and leukocyte transendothelial migration. Specifically, *PECAM1* is predominantly expressed at intercellular junctions between endothelial cells and on immune cells, including neutrophils and monocytes/macrophages. In the setting of AMI, large numbers of these immune cells are rapidly activated, and *PECAM1* expression is upregulated on both leukocytes and endothelial cells. Activated immune cells engage endothelial *PECAM1* via homophilic *PECAM1-PECAM1* interactions, thereby promoting their passage through endothelial junctions. In parallel, *PECAM1* signaling recruits *SHP-2* and triggers endothelial cytoskeletal rearrangement, leading to transient loosening of endothelial cell–cell contacts and further facilitating leukocyte transmigration (46, 47). Among leukocyte subsets, neutrophil transendothelial migration is particularly dependent on *PECAM1.* These observations suggest that transient or partial inhibition of *PECAM1* signaling might attenuate excessive leukocyte recruitment and inflammation after AMI and thereby improve outcomes.

*ZNF257* is a DNA-binding transcription factor localized in the cell nucleus, and it is believed to play a role in regulating DNA transcription. Currently, there is limited research on *ZNF257* (48). In our analysis, although *ZNF257* showed some diagnostic potential for AMI, its association with various immune cells was minimal. Therefore, the relationship between *ZNF257* and AMI needs further investigation.

In summary, a series of bioinformatics methods was employed to identify nine key biomarkers and explore their potential mechanisms in AMI. Our analysis revealed that metabolism, immunity, and inflammation are critical pathways through which these genes influence AMI development. Specifically, *LIPA, RTN2*, and *PECAM1* were found to be significantly associated with increased AMI risk, while *CFDP1, FES, XPO6*, and *HP* appeared to confer protective effects. The role of *SMARCA4* in AMI remains complex and requires further experimental investigation. *ZNF257*, *SMARCA4*, and *FES* did not show statistically significant expression changes in the mouse AMI model. Future studies integrating transcriptomic data with clinical biochemical indicators will help further evaluate the potential translational value of these genes.

### 4.1 Species/tissue limitations

For species discordance, while many cardiovascular and immune pathways are conserved across species, gene-level expression patterns may differ due to distinct cis-regulatory architectures, cell-type composition, and temporal dynamics. For tissue discordance, all TWAS, machine-learning, immune infiltration, and Bayesian colocalization analyses were based on human peripheral blood datasets, reflecting systemic immune and inflammatory responses associated with acute myocardial infarction (AMI). Experimental validation was performed in mouse left ventricular myocardium, which represents local tissue responses at the infarct site. Furthermore, we chose to validate these findings using cardiac tissue for the following reasons (1): Myocardial infarction is fundamentally a tissue-specific pathological process, and cardiac tissue allows direct assessment of molecular changes within the infarcted organ (2); Several of the identified genes are involved in endothelial function, immune cell recruitment, metabolic remodeling, and tissue repair, which are most biologically relevant within the cardiac microenvironment (3); Blood-based gene expression may reflect systemic immune responses but does not fully capture local myocardial injury and repair mechanisms (4); Many previous AMI studies use myocardial tissue to validate candidate genes due to its relevance for therapeutic targeting and mechanistic investigation. Furthermore, immune deconvolution analyses based on human peripheral blood transcriptomic data primarily capture systemic immune alterations following AMI, whereas validation experiments performed in mouse cardiac tissue reflect local myocardial injury and inflammation. We emphasize that these two layers likely represent interconnected but non-identical immune processes, and that integrating blood- and tissue-level immune profiling will be essential for fully elucidating immune mechanisms in AMI.

Specifically, we discuss issues including: (i) variability in gene expression measurements across platforms and sample sources; (ii) the need for standardized, rapid, and cost-effective assays suitable for clinical settings; (iii) challenges related to timing of sample collection relative to disease onset; and (iv) the necessity of large-scale prospective validation in diverse patient populations. These considerations highlight the translational gap between discovery-stage biomarkers and routine clinical application.

## 5 Conclusions

In this study, nine key genes (*LIPA, RTN2, PECAM1, CFDP1, FES, XPO6, SMARCA4, HP*, and *ZNF257*) were identified through the integration of multi-omics data, machine learning, and Bayesian colocalization analysis. Further immunoinfiltration analysis, single gene GSEA and GSVA analysis explored the mechanism and biological function of these 9 key genes in AMI. Finally, the expression levels of these nine genes were validated in a mouse model of myocardial infarction using PCR. Our findings provide a foundation for exploring potential regulatory targets and mechanisms of AMI and offer new insights for the development of therapeutic strategies for this condition.

## Data Availability

The original contributions presented in the study are included in the article/**Supplementary Material**. Further inquiries can be directed to the corresponding authors.
