# Supplementary material for: Identification of key biomarkers for myocardial infarction by multi-omics analysis and machine learning
Source: Front Immunol. 2026 Apr 13;17:1711521. doi: 10.3389/fimmu.2026.1711521 (PMC13111458; doi:10.3389/fimmu.2026.1711521)

Supplementary Material 1

1. ML parameters of Machine Learning

Algorithm	Key_Parameters	CV_or_Optimization
Lasso	alpha=1; lambda selected by cv.glmnet	10-fold CV
Ridge	alpha=0; lambda selected by cv.glmnet	10-fold CV
Elastic Net	alpha=0.1-0.9 (9 settings); lambda selected by cv.glmnet	10-fold CV
Stepwise GLM	direction=forward/backward/both	No internal CV
SVM	svm(..., probability=TRUE), radial kernel default	No internal CV
LDA	caret::train(method='lda')	caret CV
glmBoost	mstop selected by cvrisk; mstop>=40	k-fold CV (cvrisk)
PLS-GLM	nt=10	cv.plsRglm
Random Forest	ntree=1000; nodesize=5	OOB
GBM	n.trees=10000; depth=3; n.minobsinnode=10; shrinkage=0.001	10-fold CV
XGBoost	max.depth=2; eta=1; nrounds selected by 5-fold CV	5-fold CV
Naive Bayes	default priors	No internal CV

2. The combination of methods actually operated in this study

Method	Retained_for_Evaluation
Lasso+Stepglm[both]	TRUE
SVM	TRUE
glmBoost+SVM	TRUE
Ridge	TRUE
Lasso+SVM	TRUE
glmBoost+Ridge	TRUE
Enet[alpha=0.1]	TRUE
glmBoost+Enet[alpha=0.1]	TRUE
Enet[alpha=0.2]	TRUE
Enet[alpha=0.3]	TRUE
glmBoost+Enet[alpha=0.3]	TRUE
glmBoost+Enet[alpha=0.2]	TRUE
Enet[alpha=0.4]	TRUE
glmBoost+Enet[alpha=0.4]	TRUE
Lasso+glmBoost	TRUE
Enet[alpha=0.5]	TRUE
glmBoost	TRUE
glmBoost+Enet[alpha=0.5]	TRUE
Enet[alpha=0.6]	TRUE
glmBoost+Enet[alpha=0.6]	TRUE
glmBoost+Enet[alpha=0.7]	TRUE
glmBoost+Enet[alpha=0.8]	TRUE
Enet[alpha=0.8]	TRUE
Enet[alpha=0.9]	TRUE
Lasso	FALSE
Enet[alpha=0.7]	TRUE
glmBoost+Enet[alpha=0.9]	TRUE
glmBoost+Lasso	TRUE

glmBoost+Stepglm[forward]	TRUE
Lasso+Stepglm[forward]	TRUE
Stepglm[forward]	TRUE
Stepglm[both]+Ridge	TRUE
Stepglm[backward]+Ridge	TRUE
Stepglm[both]+Enet[alpha=0.9]	TRUE
Stepglm[backward]+Enet[alpha=0.9]	TRUE
Stepglm[both]+Enet[alpha=0.1]	TRUE
Stepglm[backward]+Enet[alpha=0.1]	TRUE
Stepglm[both]+Enet[alpha=0.8]	TRUE
Stepglm[backward]+Enet[alpha=0.8]	TRUE
Stepglm[both]+Enet[alpha=0.2]	TRUE
Stepglm[backward]+Enet[alpha=0.2]	TRUE
Stepglm[both]+Lasso	TRUE
Stepglm[backward]+Lasso	TRUE
Stepglm[both]+Enet[alpha=0.6]	TRUE
Stepglm[backward]+Enet[alpha=0.6]	TRUE
Stepglm[both]+Enet[alpha=0.7]	TRUE
Stepglm[backward]+Enet[alpha=0.7]	TRUE
Lasso+Stepglm[backward]	TRUE
Stepglm[both]	TRUE
Stepglm[backward]	TRUE
glmBoost+Stepglm[both]	TRUE
glmBoost+Stepglm[backward]	TRUE
Stepglm[both]+Enet[alpha=0.4]	TRUE
Stepglm[backward]+Enet[alpha=0.4]	TRUE
Stepglm[both]+Enet[alpha=0.3]	TRUE
Stepglm[backward]+Enet[alpha=0.3]	TRUE
Stepglm[both]+glmBoost	TRUE
Stepglm[backward]+glmBoost	TRUE
Stepglm[both]+Enet[alpha=0.5]	TRUE
Stepglm[backward]+Enet[alpha=0.5]	TRUE
Stepglm[both]+SVM	TRUE
Stepglm[backward]+SVM	TRUE
LDA	TRUE
glmBoost+LDA	TRUE
Stepglm[both]+LDA	TRUE
Stepglm[backward]+LDA	TRUE
Lasso+LDA	TRUE
NaiveBayes	TRUE
Lasso+NaiveBayes	TRUE
glmBoost+NaiveBayes	TRUE
Stepglm[both]+NaiveBayes	TRUE
Stepglm[backward]+NaiveBayes	TRUE

3. Learning curve details

Fraction	N_Models	Train_AUC_Mean	Train_AUC_SD	Validation_AUC_Mean	Validation_AUC_SD
0.2	4	1	0	0.696465497	0.101788373
0.4	6	0.910964912	0.071497955	0.78080074	0.036734896
0.6	6	0.874904215	0.0278096	0.719007247	0.059998384
0.8	7	0.858058608	0.036929711	0.730338786	0.047851462
1	10	0.860244898	0.021594713	0.770459648	0.01401103

4. Learn curve point data

Fraction	Repeat	Train_AUC	Validation_AUC
0.2	1	1	0.715762732
0.2	4	1	0.55621529
0.2	8	1	0.713783528
0.2	10	1	0.800100437
0.4	1	0.986842105	0.761313955
0.4	2	0.952631579	0.801400213
0.4	5	0.852631579	0.817854189
0.4	7	0.797368421	0.720105164
0.4	9	0.95	0.809848753
0.4	10	0.926315789	0.774282169
0.6	2	0.865517241	0.685306629
0.6	4	0.904597701	0.675410611
0.6	5	0.885057471	0.795108118
0.6	6	0.825287356	0.6560026
0.6	8	0.893103448	0.790736146
0.6	10	0.875862069	0.711479381
0.8	1	0.859615385	0.707934539
0.8	3	0.824358974	0.655529954
0.8	4	0.852564103	0.702321872
0.8	6	0.925	0.739660877
0.8	7	0.875641026	0.737681673
0.8	8	0.858333333	0.764356611
0.8	9	0.810897436	0.804885974
1	1	0.849387755	0.762052464
1	2	0.850612245	0.755789909
1	3	0.872653061	0.770737327
1	4	0.874285714	0.770737327
1	5	0.871836735	0.767990074
1	6	0.804489796	0.807751388
1	7	0.866122449	0.764799716
1	8	0.872653061	0.770737327
1	9	0.868571429	0.763263618
1	10	0.871836735	0.770737327

5. Learning curve graph

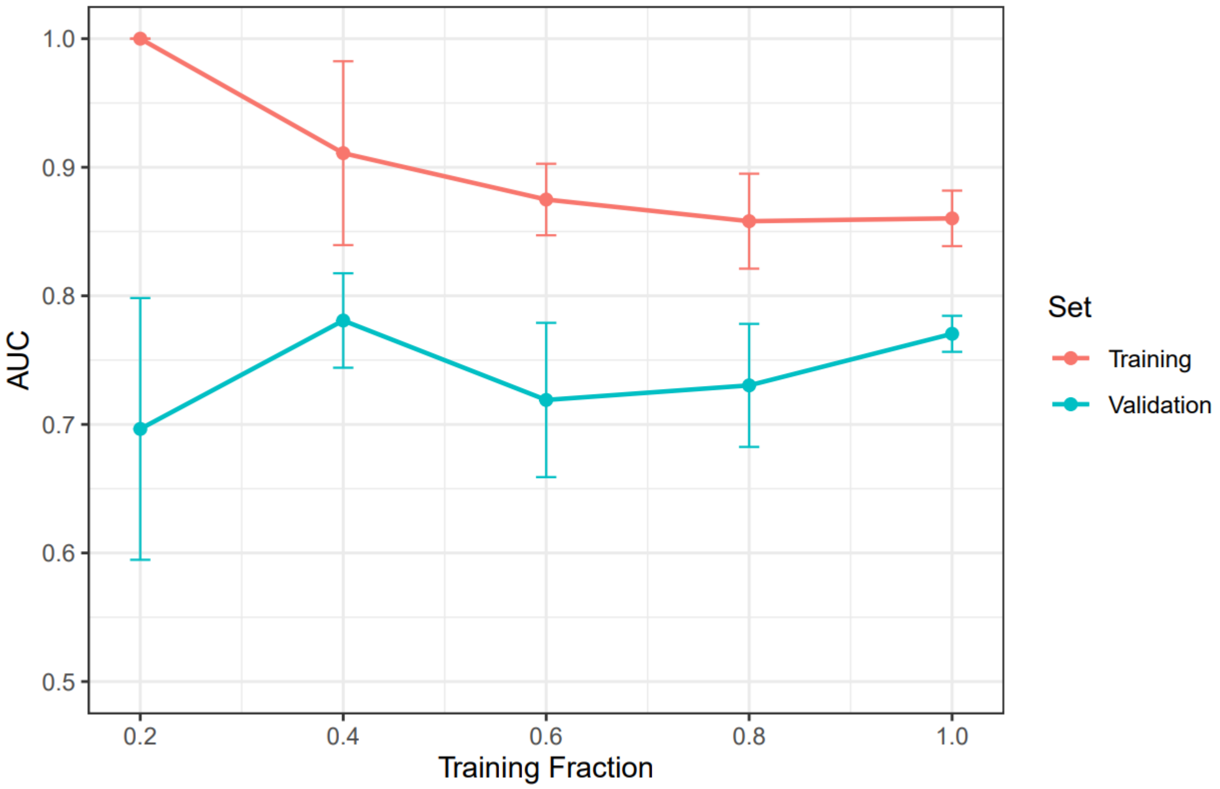

6. the complete metric table of the final model (Lasso+glmBoost):

Cohort	N	Cases	Controls	Threshold_	AUC	AUC_CI_Lo	AUC_CI_Hi	TP	TN	FP	FN	Sensitivity	Specificity	PPV	NPV	Accuracy	BalancedA	F1	MCC
GSE66360	99	49	50	0.443657	0.873878	0.798208	0.949548	40	43	7	9	0.816327	0.86	0.851064	0.826923	0.838384	0.838163	0.833333	0.677156
GSE48060	52	31	21	0.952958	0.75576	0.615328	0.896193	25	15	6	6	0.806452	0.714286	0.806452	0.714286	0.769231	0.760369	0.806452	0.520737
GSE60993	33	26	7	0.997001	0.78022	0.574095	0.986345	23	5	2	3	0.884615	0.714286	0.92	0.625	0.848485	0.799451	0.901961	0.571315

7. the analysis of the importance of 100 substitutions

Feature	Mean_Delta_AUC	SD_Delta_AUC	Median_Delta_AUC	Positive_Delta_Rate
XPO6	0.130746939	0.026139904	0.131020408	1
FES	0.086302041	0.027344158	0.086734694	1
MAT2A	0.052159184	0.016961701	0.053061224	1
CFDP1	0.048134694	0.019353082	0.048571429	1
HP	0.037795918	0.017432254	0.037755102	0.99
SMARCA4	0.018489796	0.010336474	0.017755102	0.97
FHL3	0.010171429	0.008562625	0.010408163	0.89
RTN2	0.004587755	0.003717467	0.004897959	0.88
SF3A3	0.003044898	0.001761958	0.002857143	0.95
ZNF257	0.001004082	0.001854101	0.00122449	0.67
ZNF90	0.000906122	0.001191163	0.000816327	0.7
PECAM1	0.000906122	0.002413388	0.000816327	0.59
MTAP	0	0	0	0
LIPA	0	0	0	0

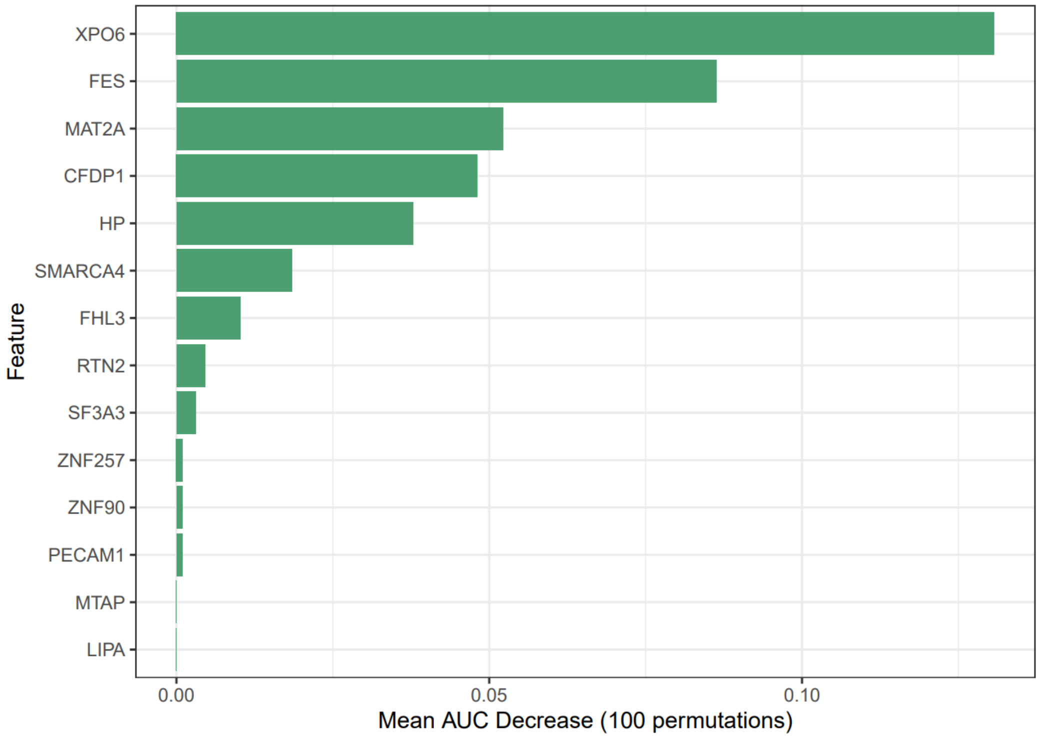

8. the calibration results of each cohort

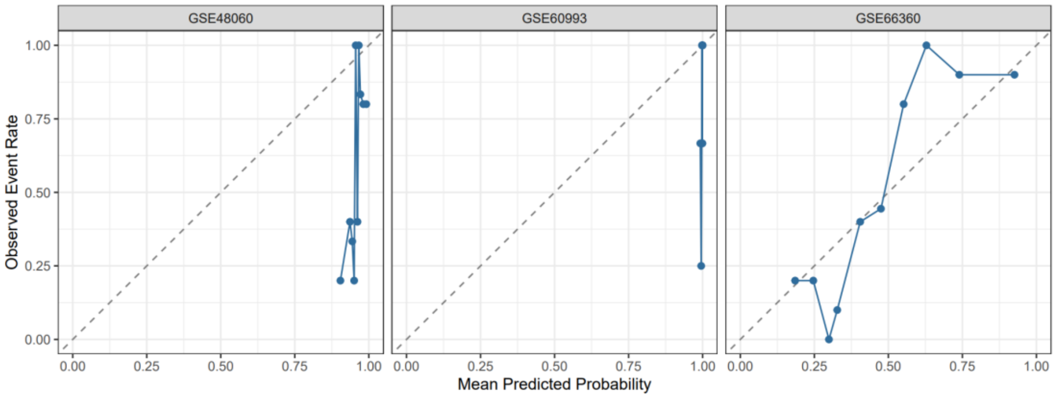

Cohort	Brier	HL_ChiSquare	HL_df	HL_p_value
GSE66360	0.160075638	15.36765788	8	0.052378683
GSE48060	0.362076564	212.0492999	8	0
GSE60993	0.210354512	1752.284308	8	0

mean_pred	obs_rate	n	cohort
0.1851187	0.2	10	GSE66360
0.246420032	0.2	10	GSE66360
0.299310718	0	10	GSE66360

0.327366352	0.1	10	GSE66360
0.404911597	0.4	10	GSE66360
0.474986928	0.444444444	9	GSE66360
0.551620676	0.8	10	GSE66360
0.628480001	1	10	GSE66360
0.73960734	0.9	10	GSE66360
0.925855841	0.9	10	GSE66360
0.903960997	0.2	5	GSE48060
0.936354022	0.4	5	GSE48060
0.944016171	0.333333333	6	GSE48060
0.950392463	0.2	5	GSE48060
0.95564416	1	5	GSE48060
0.961639722	0.4	5	GSE48060
0.966125965	1	5	GSE48060
0.97143547	0.833333333	6	GSE48060
0.981387605	0.8	5	GSE48060
0.991337744	0.8	5	GSE48060
0.992149312	0.666666667	3	GSE60993
0.995163045	0.25	4	GSE60993
0.997037882	0.666666667	3	GSE60993
0.997876429	1	3	GSE60993
0.998204803	1	4	GSE60993

0.998665363	0.666666667	3	GSE60993
0.999134712	0.666666667	3	GSE60993
0.999323124	1	4	GSE60993
0.999745817	1	3	GSE60993
0.999834452	1	3	GSE60993

9. Categorical distribution in validation sets

Cohort	Cases	Controls	Case_Control_Ratio
GSE66360	49	50	0.98
GSE48060	31	21	1.476
GSE60993	26	7	3.714

10. Unbalanced robust metrics in validation sets

Cohort	N	Cases	Controls	Threshold_	AUC	AUC_CI_Lc	AUC_CI_Hi	TP	TN	FP	FN	Sensitivity	Specificity	PPV	NPV	Accuracy	BalancedA	F1	MCC
GSE66360	99	49	50	0.443657	0.873878	0.798208	0.949548	40	43	7	9	0.816327	0.86	0.851064	0.826923	0.838384	0.838163	0.833333	0.677156
GSE48060	52	31	21	0.952958	0.75576	0.615328	0.896193	25	15	6	6	0.806452	0.714286	0.806452	0.714286	0.769231	0.760369	0.806452	0.520737
GSE60993	33	26	7	0.997001	0.78022	0.574095	0.986345	23	5	2	3	0.884615	0.714286	0.92	0.625	0.848485	0.799451	0.901961	0.571315

11. sensitivity analysis under a fixed training threshold

Cohort	N	Cases	Controls	Threshold_	TP	TN	FP	FN	Sensitivity	Specificity	PPV	NPV	Accuracy	BalancedA	F1	MCC
GSE66360	99	49	50	0.443657	40	43	7	9	0.816327	0.86	0.851064	0.826923	0.838384	0.838163	0.833333	0.677156
GSE48060	52	31	21	0.443657	31	0	21	0	1	0	0.596154	NA	0.596154	0.5	0.746988	NA
GSE60993	33	26	7	0.443657	26	0	7	0	1	0	0.787879	NA	0.787879	0.5	0.881356	NA

12. Immune deconvolution of key genes by EPIC

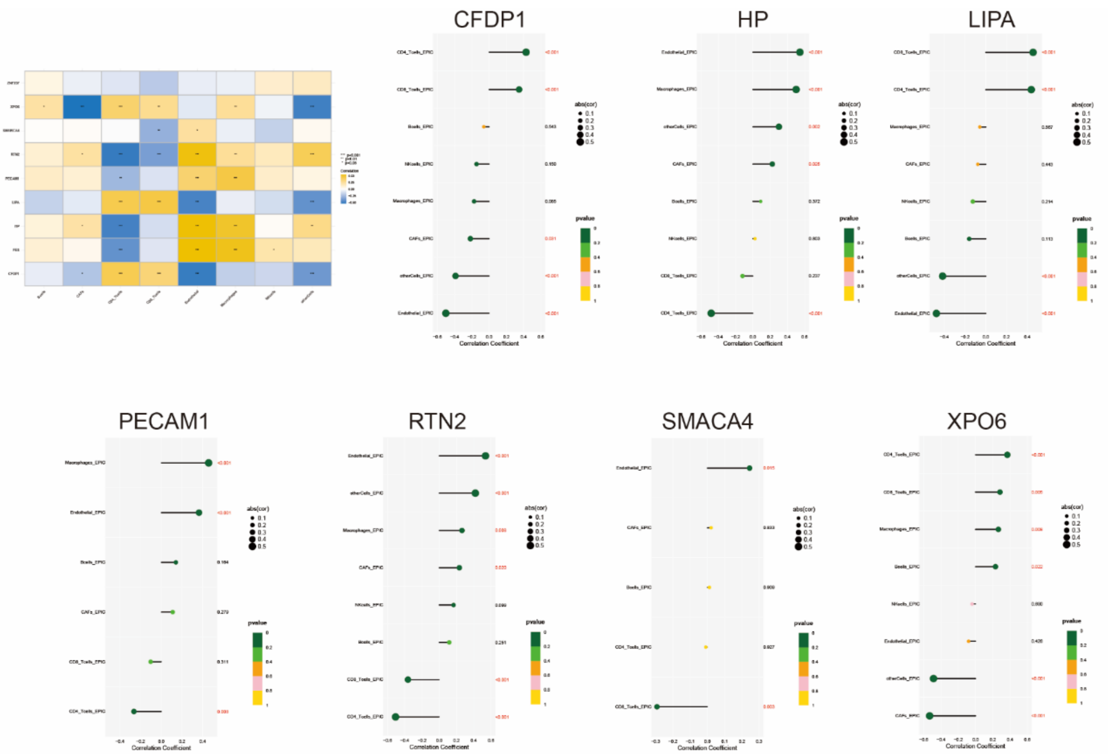

13. PCA plots before and after batch correction

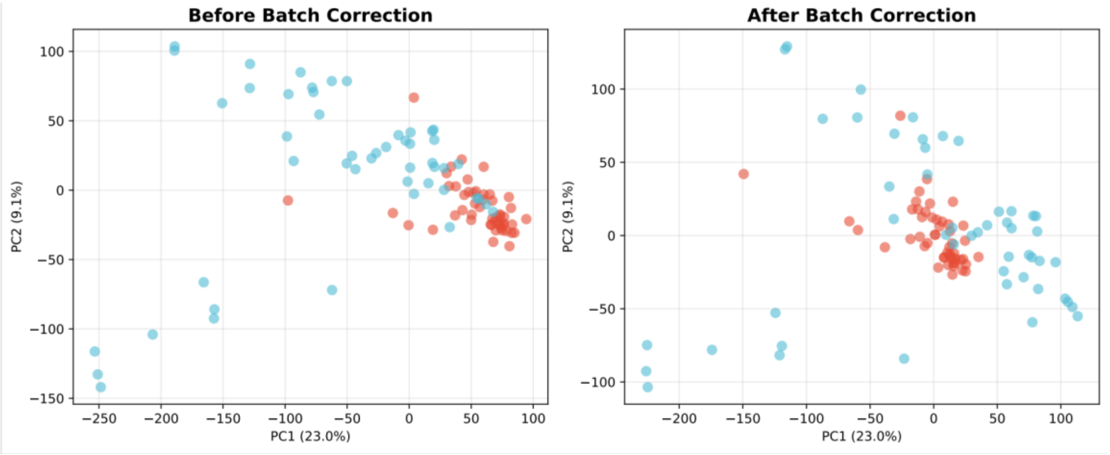

Supplement: Supplementary file 1 [file SupplementaryFile1.pdf]
