# Supplementary material for: Identification of key biomarkers for myocardial infarction by multi-omics analysis and machine learning
Source: Front Immunol. 2026 Apr 13;17:1711521. doi: 10.3389/fimmu.2026.1711521 (PMC13111458; doi:10.3389/fimmu.2026.1711521)
Supplement: Supplementary file 2 [file SupplementaryFile2.pdf]

Supplementary Material 2

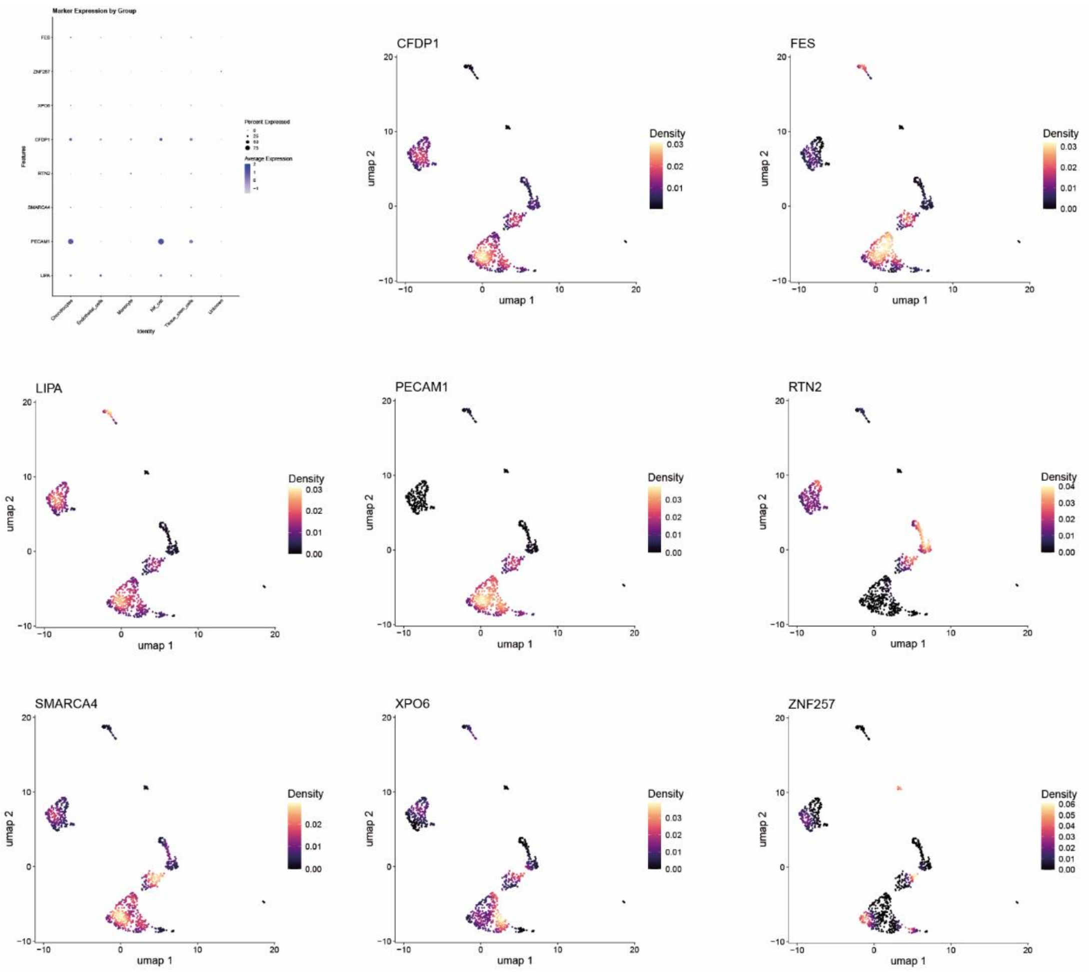

FigureS1: Single-cell RNA sequencing analysis of eight genes for AMI.

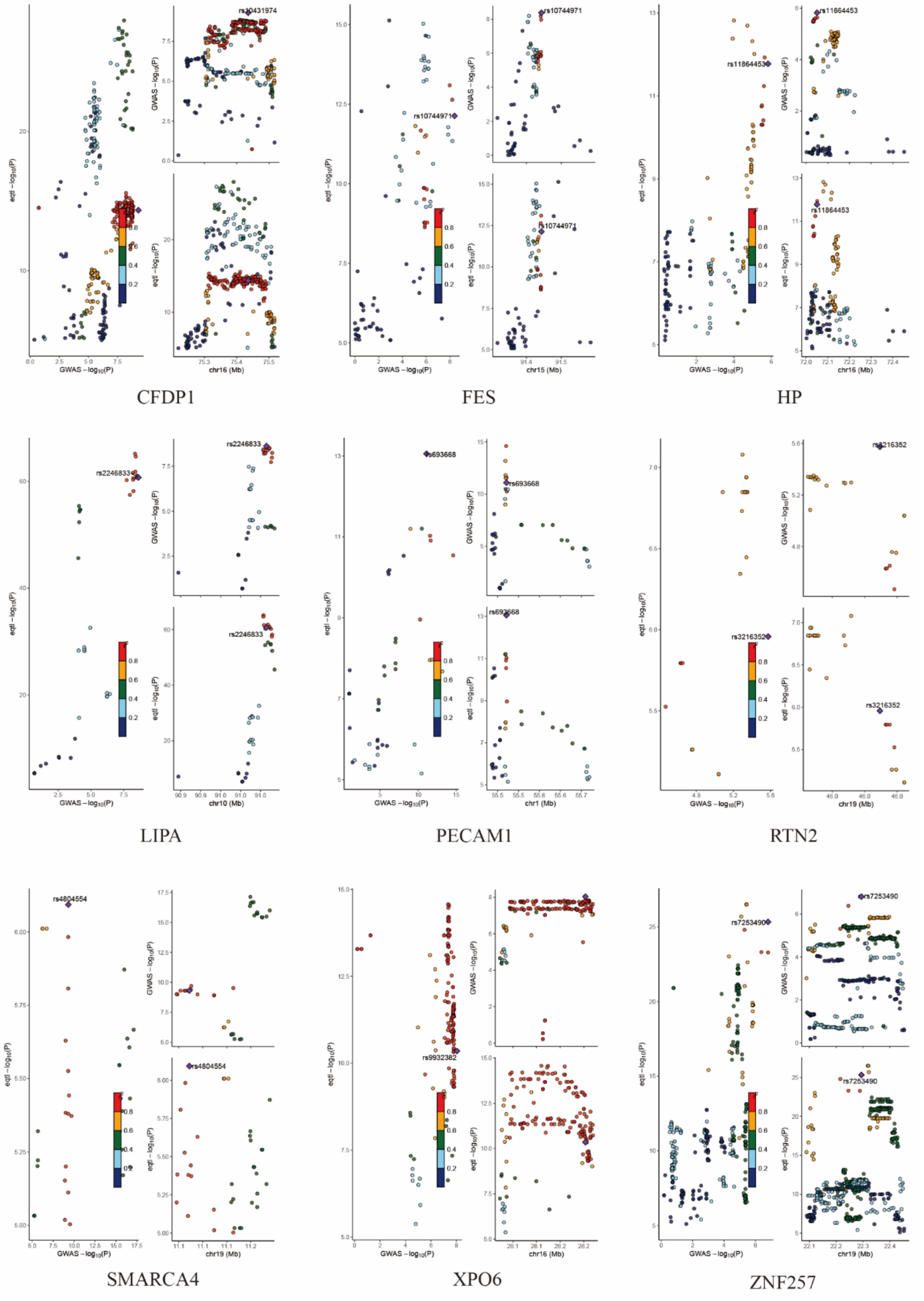

FigureS2: The regional association plots of key genes for GWAS and eQTL signals

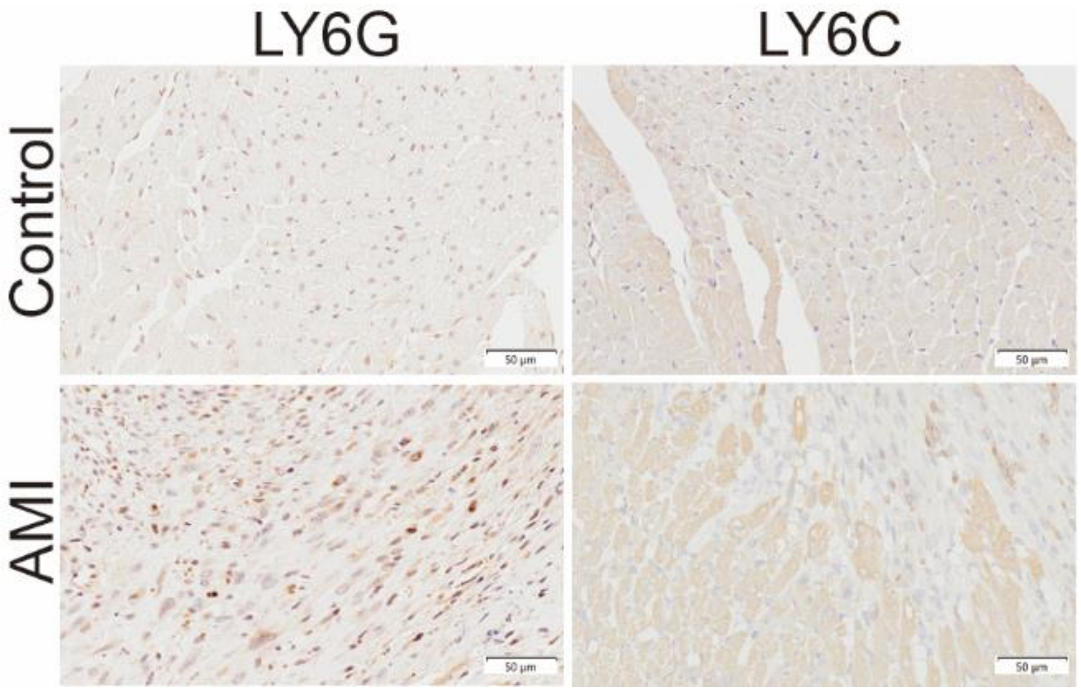

FigureS3: The immunohistochemistry representative graph of LY6G and LY6C in the heart.

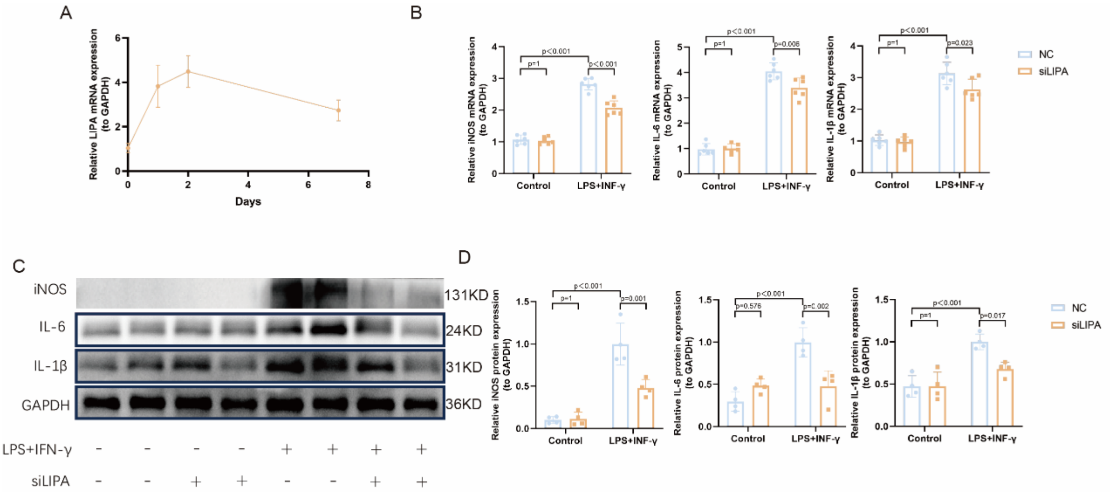

FigureS4: A and B. Verification of the LIPA by RT-qPCR. C and D. The protein expression levels of LIPA in the Macrophage was analyzed by Western blot.
